# Tumor suppressor p53 mediates interleukin-6 expression to enable cancer cell evasion of genotoxic stress

**DOI:** 10.1038/s41420-023-01638-0

**Published:** 2023-09-11

**Authors:** Trinh T. T. Phan, Nam V. Truong, Wen-Guey Wu, Yi-Chun Su, Tzu-Sheng Hsu, Lih-Yuan Lin

**Affiliations:** 1 Institute of Molecular and Cellular Biology, College of Life Sciences and Medicine, National Tsing Hua University, Hsinchu, 300044 Taiwan ROC; 2 Institute of Bioinformatics and Structural Biology, College of Life Sciences and Medicine, National Tsing Hua University, Hsinchu, 300044 Taiwan ROC

**Keywords:** Cell death, Cell invasion, Calcium signalling, Metastasis, Tumour-suppressor proteins

## Abstract

The tumor suppressor p53 primarily functions as a mediator of DNA damage-induced cell death, thereby contributing to the efficacy of genotoxic anticancer therapeutics. Here, we show, on the contrary, that cancer cells can employ genotoxic stress-induced p53 to acquire treatment resistance through the production of the pleiotropic cytokine interleukin (IL)-6. Mechanistically, DNA damage, either repairable or irreparable, activates p53 and stimulates Caspase-2-mediated cleavage of its negative regulator mouse double minute 2 (MDM2) creating a positive feedback loop that leads to elevated p53 protein accumulation. p53 transcriptionally controls the major adenosine triphosphate (ATP) release channel pannexin 1 (Panx1), which directs IL-6 induction via a mechanism dependent on the extracellular ATP-activated purinergic P2 receptors as well as their downstream intracellular calcium (iCa^2+^)/PI3K/Akt/NF-ĸB signaling pathway. Thus, p53 silencing impairs Panx1 and IL-6 expression and renders cancer cells sensitive to genotoxic stress. Moreover, we confirm that IL-6 hampers the effectiveness of genotoxic anticancer agents by mitigating DNA damage, driving the expression of anti-apoptotic Bcl-2 family genes, and maintaining the migratory and invasive properties of cancer cells. Analysis of patient survival and relevant factors in lung cancer and pan-cancer cohorts supports the prognostic and clinical values of Panx1 and IL-6. Notably, IL-6 secreted by cancer cells during genotoxic treatments promotes the polarization of monocytic THP-1-derived macrophages into an alternative (M2-like) phenotype that exhibits impaired anti-survival activities but enhanced pro-metastatic effects on cancer cells as compared to nonpolarized macrophages. Our study reveals the precise mechanism for genotoxic-induced IL-6 and suggests that targeting p53-mediated IL-6 may improve the responsiveness of cancer cells to genotoxic anticancer therapy.

## Introduction

Genotoxic agents remain an important therapeutic approach to cancer treatments [1–3]. These agents exert cytotoxic effects by interfering with components of the DNA replication machinery and repair, leading to extensive DNA lesions and ultimately cell death or cell cycle blockage [4]. Although cancer cells may have favorable initial responses to these drugs, they can deploy an arsenal of mechanisms to impair treatment efficacy and prevent the complete eradication of the tumors [5]. Thus, an advanced understanding of the molecular basis of cellular responses to the treatments would benefit the development of therapeutic strategies to overcome drug resistance and enhance the effectiveness of these genotoxic anticancer agents.

Recently, interleukin (IL)-6, an inflammation-associated cytokine with multifaceted effects, has emerged as a critical therapeutic target in cancer treatments [6, 7]. IL‑6 is produced by multiple cell lineages in the tumor microenvironment [7] and exerts its oncogenic effects via both autocrine and paracrine mechanisms [8–10]. IL‑6 acts directly on cancer cells to potentiate cancer progression, metastasis, angiogenesis, stemness, and chemoresistance [8, 10–13]. It also influences stromal and tumor-infiltrating immune cells, contributing to a highly immunosuppressive and pro-tumorigenic tumor microenvironment [9, 13, 14]. Importantly, IL-6 is one of the most predominantly upregulated inflammatory cytokines in response to genotoxic treatments [9, 12, 15]. However, the molecular mechanisms by which genotoxic stress triggers IL-6 induction are not well understood.

The tumor suppressor p53, encoded by the tumor protein p53 (*TP53*) gene, is a master regulator of cellular responses to diverse stresses, including genotoxic stress [16, 17]. p53 contributes to cancer cell sensitivity to genotoxic treatments by regulating multiple target genes involved in cell cycle arrest, cellular senescence, programmed cell death, metabolism, and anti-metastasis [17–19]. p53 also provides benefits to cancer cells by controlling many pro-survival cellular processes, such as metabolic stress responses, redox homeostasis, and DNA repair [18, 20, 21]. These unexpected effects serve as a considerable obstacle hampering the efficacy of p53-based anticancer therapy. Nonetheless, current knowledge of the oncogenic roles of p53, particularly in the context of genotoxic treatments, remains scarce.

In this study, we identify p53 as a key regulator mediating IL-6 induction in cancer cells during genotoxic stress. Under both reversible and irreversible genotoxic conditions, increased p53 abundance via Caspase-2-mediated cleavage of its primary negative regulator mouse double minute 2 (MDM2) leads to elevated expression of the integral membrane channel protein pannexin 1 (Panx1), which is implicated in the release of intracellular adenosine triphosphate (ATP) into the extracellular space and subsequent activation of the ATP-activated purinergic P2 receptors (P2Rs). Panx1 and P2Rs are further identified as important contributors to genotoxic stress-induced IL-6 expression, acquired via the intracellular calcium (iCa^2+^)/PI3K/Akt/NF-ĸB signaling axis. Moreover, we confirm that IL-6 favors cell survival and motility in both autocrine and paracrine manners. Thus, the abolishment of p53-mediated IL-6 expression effectively enhances cellular responses to genotoxic treatments.

## Results

### Downregulation of IL-6 renders cancer cells sensitive to genotoxic treatments

**Fig. 1:**
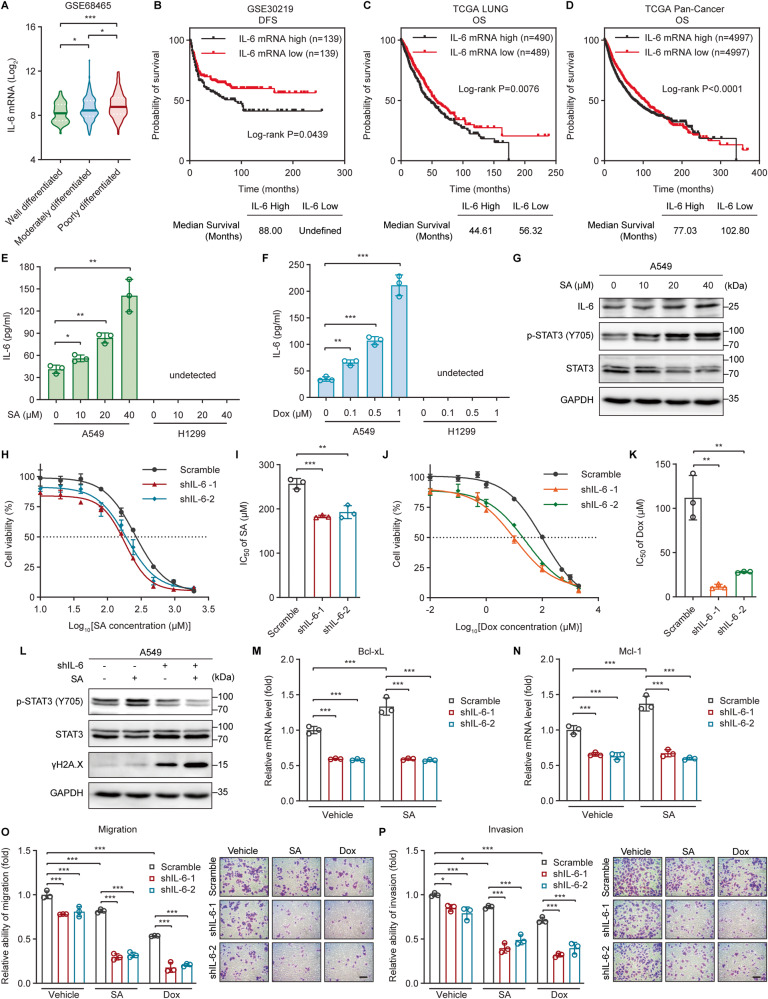
Downregulation of IL-6 renders cancer cells sensitive to genotoxic treatments. **A** IL-6 mRNA levels in lung adenocarcinomas with different histological grades were investigated using the GSE68465 dataset and their log_2_-transformed values were compared among well (*n* = 60), moderately (*n* = 209), and poorly (*n* = 167) differentiated tumors. **B** Kaplan–Meier analysis of the relationship between IL-6 gene expression and disease-free survival (DFS) in lung cancer patients from the GSE30219 cohort. **C**, **D** Kaplan–Meier analysis of the relationship between IL-6 gene expression and overall survival (OS) in cancer patients from The Cancer Genome Atlas (TCGA) (**C**) lung cancer (LUNG) or (**D**) Pan-Cancer datasets. **E**, **F** The concentration of IL-6 present in the culture supernatants of A549 or H1299 cells treated with the indicated concentrations of (**E**) sodium arsenite (SA) or (**F**) doxorubicin (Dox) for 24 h was determined by ELISA. **G** Western blot analysis of IL-6, phosphorylated STAT3 (Y705), and total STAT3 protein levels in A549 cells treated with increasing concentrations of SA for 24 h. **H**, **J** Dose-response curves showing the survival of control (scramble) and IL-6-silenced (shIL-6-1 and shIL-6-2) A549 cells in response to increasing concentrations of (**H**) SA or (**J**) Dox treatments for 24 h. **I**, **K** The IC_50_ values of (**I**) SA or (**K**) Dox against scramble, shIL-6-1, and shIL-6-2 A549 cells were calculated from the nonlinear regression curves in Fig. 1H or J, respectively. Cell viability was measured by MTT assays. **L** Phosphorylated STAT3 (Y705), total STAT3, and γH2A.X protein levels in control (scramble) and IL-6-silenced (shIL-6) A549 cells untreated or treated with 20 µM SA for 24 h were determined by western blot analysis. **M**, **N** The mRNA levels of the anti-apoptotic genes (**M**) Bcl-xL and (**N**) Mcl-1 in scramble, shIL-6-1, and shIL-6-2 A549 cells untreated or treated with 20 µM SA for 24 h were measured by qRT-PCR. **O**, **P** The (**O**) migratory and (**P**) invasive abilities of scramble, shIL-6-1, and shIL-6-2 A549 cells untreated or treated with 20 µM SA or 0.1 µM Dox for 24 h were measured with transwell migration and invasion assays, respectively. Scale bar: 100 µm. Error bars represent mean ± SD, *n* = 3. Statistical analysis was performed using one-way ANOVA with Tukey’s multiple comparisons test (**A**), log-rank test (**B**–**D**), unpaired two-tailed Student’s *t* test (**E**, **F**, **I**, **K**), or two-way ANOVA with Tukey’s multiple comparisons test (**M**–**P**). *p ≤ 0.05; **p ≤ 0.01; ***p ≤ 0.001. The full length uncropped original western blots related to this figure are provided in the Supplemental Material file.

IL-6 is a key mediator of multiple cellular processes regulating tumor growth and treatment resistance [7]. We hypothesized that IL-6 expression levels can predict the clinical outcome of cancer patients. Indeed, elevated IL-6 expression was associated with poorly differentiated (high-grade) histology, whereas IL-6 was expressed at relatively low levels in well differentiated (low-grade) lung adenocarcinomas (Fig. 1A). Consistently, lung cancer patients with high IL-6 expression displayed significantly shorter disease-free survival (DFS) (Fig. 1B) and overall survival (OS) (Fig. 1C) than those having lower IL-6 expression. Moreover, we also observed a similar prognostic value of IL-6 in tumors across The Cancer Genome Atlas (TCGA) Pan-Cancer dataset (Fig. 1D), suggesting that the role of IL-6 in promoting cancer progression and hindering patient survival is common among a wide range of cancers.

To gain a comprehensive understanding of the roles of IL-6 signaling in cancer cell responses to genotoxic treatments, we started out by investigating changes in the levels of IL-6 and its receptor (IL-6Rα) as well as in the phosphorylation of its downstream effector STAT3 (signal transducer and activator of transcription 3) following the treatments of cancer cells with DNA-damaging agents, such as sodium arsenite (SA) [22], doxorubicin (Dox) [23], or cisplatin (CisPt) [24]. IL-6 gene expression was dose-dependently induced by SA, Dox, or CisPt in human non-small cell lung cancer (NSCLC) A549 cells (Fig. S1A–C). Moreover, Dox elevated IL-6 expression in primary human liposarcoma cell cultures (Fig. S1D). A gradient of increased IL-6 secretion into the culture medium was also observed upon SA, Dox, or CisPt treatment (Fig. 1E, F and S1E). Consistently, SA-augmented IL-6 protein expression was accompanied by the enhanced phosphorylation of tyrosine (Y)705 on STAT3 (Fig. 1G), suggesting the activation of the IL-6/STAT3 signaling pathway in response to SA treatment. In contrast, the cell surface expression of IL-6Rα was not altered by SA or CisPt (Fig. S1F, G). Furthermore, IL-6 was expressed at undetectable levels and was not induced by genotoxic agents in p53-null NSCLC H1299 cells (Figs. 1E, F and S1A–C, E). These results underscore genotoxic agents as critical modulators of the IL-6 signaling pathway, potentially via regulating the levels of IL-6, but not membrane-bound IL-6Rα, in cancer cells, especially in p53 wild-type (WT) cells.

We next suppressed IL-6 expression in A549 cells with either an IL-6 short hairpin (sh)RNA system (Fig. S1H, I) or a small interfering (si)RNA (Fig. S1J, K), and then examined cellular responses to genotoxic treatments. We found that IL-6 knockdown aggravated cell death induced by SA (Fig. 1H, I), Dox (Fig. 1J, K), or CisPt (Fig. S1L, M) treatment. The half inhibitory concentration (IC_50_) values of SA, Dox, and CisPt against IL-6-silenced cells were markedly decreased as compared to those against control cells (Figs. 1I, K, S1M, and Table S1). Moreover, similar results were observed in human breast cancer MCF-7 (Fig. S2A–G and Table S1) and cervical cancer HeLa (Fig. S2H–N and Table S1) cells. Consistently, pretreating A549 cells with recombinant human IL-6 significantly augmented cell tolerance to SA (Fig. S3A, B and Table S1). Especially, although the levels of IL-6 appear to be undetectable in p53-null NSCLC H1299 cells (Figs. 1E, F and S1A–C, E), IL-6 pre-treatment also enhanced the tolerance of H1299 cells to SA (Fig. S3C, D and Table S1) or CisPt (Fig. S3E, F and Table S1). Furthermore, treatment with exogenous IL-6 could partially mitigate the sensitivity of IL-6-silenced cells to SA (Fig. S3G, H and Table S1) or Dox (Fig. S3I, J and Table S1). These results suggest that the levels of IL-6 expressed in cancer cells are inversely correlated with the sensitivity of cancer cells to genotoxic agents.

To decipher the downstream targets of IL-6 signaling in genotoxic tolerance, the expression of DNA double-strand breaks indicator γH2A.X (phosphorylated histone H2A member X) and several anti-apoptotic genes, including Bcl-2, Bcl-xL, and Mcl-1 was evaluated. In line with the reduced STAT3 phosphorylation following IL-6 silencing, γH2A.X protein levels were dramatically upregulated in both control and SA-treated A549 cells (Fig. 1L). Moreover, the gene expression of Bcl-xL and Mcl-1, but not Bcl-2, was dose-dependently increased upon SA treatment (Fig. S3K–M), whereas these effects could be diminished by IL-6 depletion (Fig. 1M, N). These results suggest that IL-6 signaling may modulate DNA damage repair and anti-apoptotic functions to allow cancer cells to endure genotoxic stress.

IL-6 is an important mediator of cell motility and invasiveness [7, 11, 13]. Because there was no significant difference in cell proliferation between control and IL-6-depleted cells in either the presence or absence of 20 µM SA at 24 h post-treatment (Fig. S3N), we chose the condition of 24-h-treatment with a non-toxic concentration of SA (20 µM) or Dox (0.1 µM) to define the role of IL-6 in cell migration and invasion under genotoxic stress. As expected, IL-6 silencing heightened the anti-migratory (Fig. 1O) and -invasive (Fig. 1P) activities of SA and Dox. Collectively, these data support the notion that IL-6 confers genotoxic resistance and is an important modulator of invasive cell migration under genotoxic conditions.

### Panx1 contributes to genotoxic stress-induced IL-6 and has clinical relevance

**Fig. 2:**
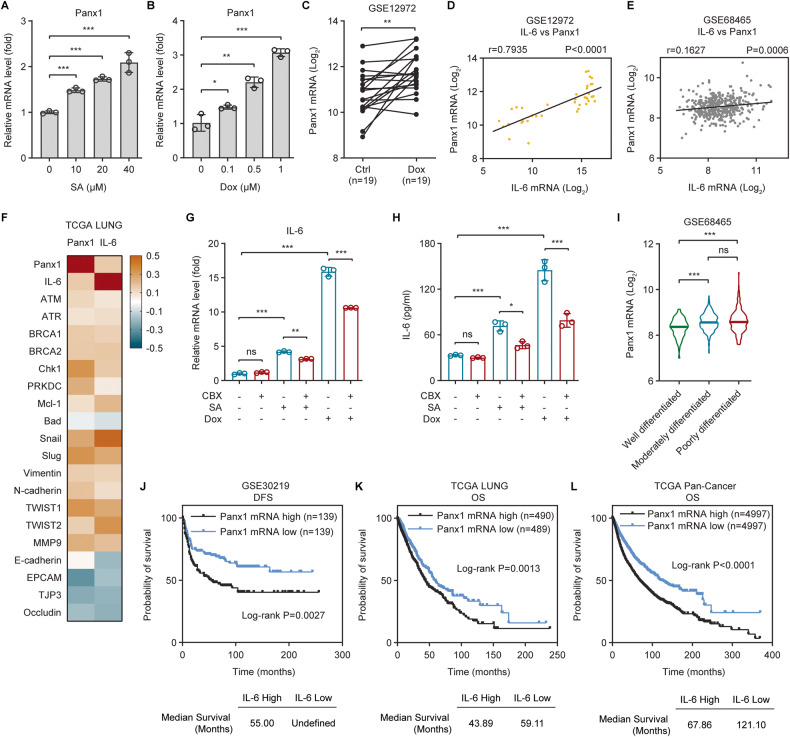
Panx1 contributes to genotoxic stress-induced IL-6 and has clinical relevance. **A**, **B** Panx1 gene expression in A549 cells treated with increasing concentrations of (**A**) SA or (**B**) Dox for 24 h was measured by qRT-PCR. **C** Panx1 mRNA levels in primary human liposarcoma cell cultures were analyzed using the GSE12972 dataset and their log_2_-transformed values were compared between control (*n* = 19) and paired Dox-treated (*n* = 19) cells. **D**, **E** Pearson’s correlation analysis of IL-6 and Panx1 gene expression in (**D**) primary human liposarcoma cell cultures or (**E**) lung adenocarcinomas using the (**D**) GSE12972 (*n* = 38) or (**E**) GSE68465 (*n* = 443) dataset, respectively. **F** Heatmap of Pearson’s correlation coefficients (*r*) between IL-6 and Panx1 or between IL-6 or Panx1 versus factors relevant to DNA damage repair (ATM, ATR, BRCA1, BRCA2, Chk1, and PRKDC), anti-apoptosis (Mcl-1), pro-apoptosis (Bad), EMT stimulation (Snail, Slug, Vimentin, N-cadherin, TWIST1, TWIST2, and MMP9), or EMT suppression (E-Cadherin, EPCAM, TJP3, and Occludin) in the TCGA LUNG cohort (*n* = 994). **G** qRT-PCR analysis of IL-6 gene expression in A549 cells untreated or treated with 20 µM SA or 0.5 µM Dox in the absence or presence of the Panx1 inhibitor CBX (50 µM) for 24 h. **H** The concentration of IL-6 present in the culture supernatants of A549 cells untreated or treated with 20 µM SA or 0.5 µM Dox in the absence or presence of 50 µM CBX for 24 h was determined by ELISA. **I** Panx1 mRNA levels in lung adenocarcinomas with different histological grades were investigated using the GSE68465 dataset and their log_2_-transformed values were compared among well (*n* = 60), moderately (*n* = 209), and poorly (*n* = 167) differentiated tumors. **J** Kaplan–Meier analysis of the relationship between Panx1 gene expression and disease-free survival (DFS) in lung cancer patients from the GSE30219 cohort. **K**, **L** Kaplan-Meier analysis of the relationship between Panx1 gene expression and overall survival (OS) in cancer patients from The Cancer Genome Atlas (TCGA) (**K**) lung cancer (LUNG) or (**L**) Pan-Cancer datasets. Error bars represent mean ± SD, *n* = 3. Statistical analysis was performed using unpaired two-tailed Student’s *t* test (**A**, **B**), paired two-tailed Student’s *t* test (**C**), two-way ANOVA with Tukey’s multiple comparisons test (**G**, **H**), one-way ANOVA with Tukey’s multiple comparisons test (**I**), or log-rank test (**J**–**L**). *p ≤ 0.05; **p ≤ 0.01; ***p ≤ 0.001; ns not significant.

Next, we focused on the molecular mechanisms underlying the induction of IL-6 by genotoxic stress. Accumulating evidence shows that the major ATP release channel pannexin 1 (Panx1) is implicated in inflammatory cytokines production and inflammation [25–27]. Panx1 gene expression was dose-dependently amplified upon SA or Dox treatment in A549 cells (Fig. 2A, B). Dox also heightened Panx1 mRNA levels in primary human liposarcoma cell cultures (Fig. 2C). Notably, Panx1 transcription was tightly correlated with IL-6 gene expression in both primary human liposarcomas (Fig. 2D) and clinical tumor tissues (Fig. 2E, F). Furthermore, both Panx1 and IL-6 mRNA levels displayed direct correlations with the expression of factors relevant to DNA damage repair (ATM, ATR, BRCA1, BRCA2, Chk1, and PRKDC), anti-apoptosis (Mcl-1), and epithelial-mesenchymal transition (EMT) (Snail, Slug, Vimentin, N-cadherin, TWIST1, TWIST2, and MMP9), but not with that of genes related to pro-apoptosis (Bad) and EMT inhibition (E-Cadherin, EPCAM, TJP3, and Occludin) (Fig. 2F). Inhibition of Panx1 channel function with carbenoxolone (CBX), a potent and widely used Panx1 inhibitor [28], led to significant decreases in both SA/Dox-induced IL-6 gene expression and protein secretion (Fig. 2G, H), suggesting that Panx1 is critical for IL-6 induction upon genotoxic treatments.

We then investigated the clinical values of Panx1. Similar to IL-6, Panx1 mRNA was strongly expressed in poorly differentiated lung adenocarcinomas but was only weakly expressed in their well differentiated counterparts (Fig. 2I). Elevated Panx1 expression was correlated with impaired DFS (Fig. 2J) and OS (Fig. 2K) in lung cancer patients. Moreover, Panx1 mRNA levels are inversely correlated with the OS of cancer patients across the TCGA Pan-Cancer dataset (Fig. 2L). Altogether, these results imply that Panx1 contributes to genotoxic-induced IL-6 and is linked to poor clinical outcomes in cancer patients.

### ATP-activated purinergic P2 receptors and the iCa^2+^/PI3K/Akt/NF-ĸB signaling pathway contribute to the genotoxic stress-induced IL-6 expression

**Fig. 3:**
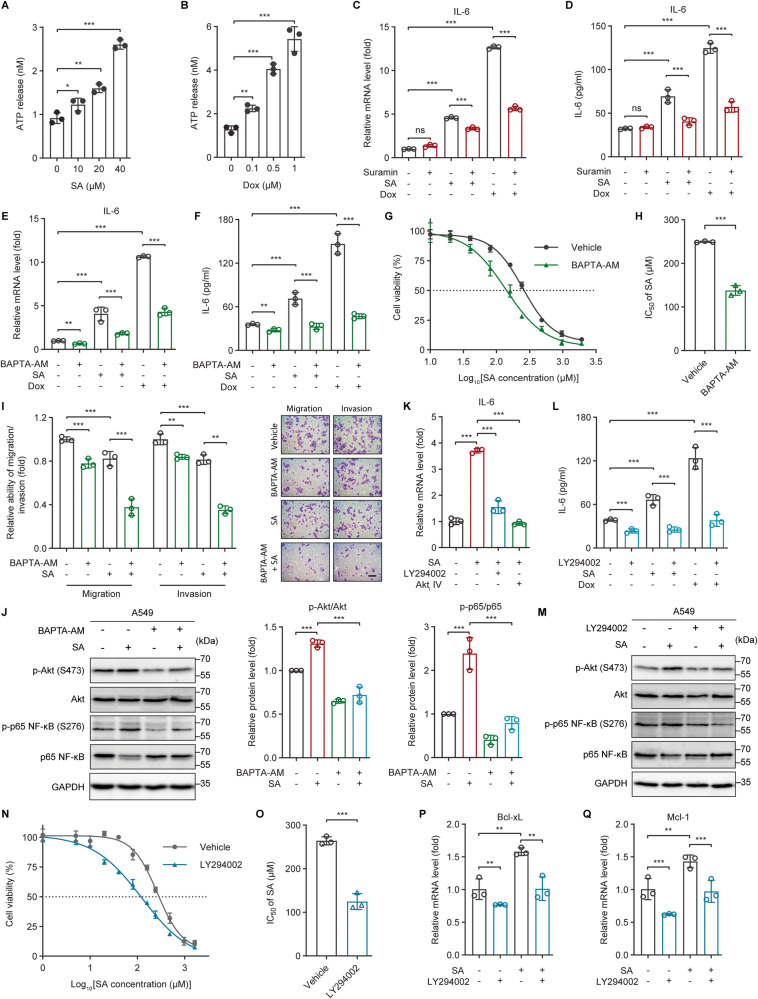
ATP-activated purinergic P2 receptors and the iCa^2+^/PI3K/Akt/NF-ĸB signaling pathway contribute to genotoxic stress-induced IL-6 expression. **A**, **B** The concentration of ATP released into the culture medium of A549 cells treated with the indicated concentrations of (**A**) SA or (**B**) Dox for 24 h was measured by a bioluminescence assay. **C**, **E** qRT-PCR analysis of IL-6 gene expression in A549 cells untreated or treated with 20 µM SA or 0.5 µM Dox in the absence or presence of (**C**) the broad-spectrum P2 receptor antagonist Suramin (50 µM) or (**E**) the intracellular calcium chelator BAPTA-AM (10 µM) for 24 h. **D**, **F** The concentration of IL-6 present in the culture supernatants of A549 cells untreated or treated with 20 µM SA or 0.5 µM Dox in the absence or presence of (**D**) 50 µM Suramin or (**F**) 10 µM BAPTA-AM for 24 h was determined by ELISA. **G** Dose-response curves showing the survival of control and 10 µM BAPTA-AM-pretreated A549 cells in response to increasing concentrations of SA treatment for 24 h. **H** The IC_50_ values of SA against control and 10 µM BAPTA-AM-pretreated A549 cells were calculated from the nonlinear regression curves in Fig. 3G. Cell viability was measured by MTT assays. **I** The migratory and invasive abilities of A549 cells untreated or treated with 20 µM SA in the absence or presence of 10 µM BAPTA-AM for 24 h were measured with transwell assays. Scale bar: 100 µm. **J**, **M** Western blot analysis of phosphorylated Akt (S473), total Akt, phosphorylated p65 NF-ĸB (S276), and total p65 NF-ĸB protein levels in A549 cells untreated or treated with 20 µM SA in the absence or presence of (**J**) 10 µM BAPTA-AM or (**M**) 10 µM LY294002 for 24 h. **K** IL-6 gene expression in A549 cells untreated or treated with 20 µM SA in the absence or presence of the PI3K inhibitor LY294002 (10 µM) or the Akt Inhibitor IV (Akt_i_ IV, 10 µM) for 24 h was measured by qRT-PCR. **L** The concentration of IL-6 protein present in the culture supernatants of A549 cells untreated or treated with 20 µM SA or 0.5 µM Dox in the absence or presence of 10 µM LY294002 for 24 h was determined by ELISA. **N** Dose-response curves showing the survival of control and 10 µM LY294002-pretreated A549 cells in response to increasing concentrations of SA treatment for 24 h. **O** The IC_50_ values of SA against control and 10 µM LY294002-pretreated A549 cells were calculated from the nonlinear regression curves in Fig. 3N. Cell viability was measured by the MTT assay. **P**, **Q** The mRNA levels of the anti-apoptotic genes (**P**) Bcl-xL and (**Q**) Mcl-1 in A549 cells untreated or treated with 20 µM SA in the absence or presence of 10 µM LY294002 for 24 h were measured by qRT-PCR. Error bars represent mean ± SD, *n* = 3. Statistical analysis was performed using unpaired two-tailed Student’s *t* test (**A**, **B**, **H**, **O**), two-way ANOVA with Tukey’s multiple comparisons test (**C**–**F**, **I**, **J**, **L**, **P**, **Q**), or one-way ANOVA with Tukey’s multiple comparisons test (**K**). *p ≤ 0.05; **p ≤ 0.01; ***p ≤ 0.001; ns not significant. The full length uncropped original western blots related to this figure are provided in the Supplemental Material file.

To date, the major function of Panx1 has been attributed to its role in mediating the release of intracellular ATP into the extracellular space where it can be recognized by, and activates, the plasma membrane-localized purinergic P2X (ionotropic) and P2Y (metabotropic) receptors (P2Rs) [29–32]. In line with increased Panx1 expression upon genotoxic treatments (Fig. 2A–C), the extracellular ATP concentration was dose-dependently elevated in SA- or Dox-treated A549 cells compared to untreated cells (Fig. 3A, B). Moreover, similar to the phenomenon seen upon Panx1 inhibition, disrupting the activity of P2Rs with the non-selective P2 receptor antagonist Suramin significantly attenuated SA/Dox-induced IL-6 gene expression and protein production (Fig. 3C, D), indicating the regulatory effect of P2Rs on IL-6 expression under genotoxic stress.

Panx1 can act via P2Rs to rise the iCa^2+^ concentration [32–34]. Panx1 also promotes direct Ca^2+^ uptake or release by forming Ca^2+^-permeable channels in the plasma and the endoplasmic reticulum (ER) membranes, respectively [25, 35, 36]. We observed that iCa^2+^ sequestration with the cell-permeable Ca^2+^ chelator BAPTA-AM led to marked decreases in IL-6 gene expression and protein secretion under both basal and genotoxic conditions (Fig. 3E, F). Accordingly, BAPTA-AM-pretreated cells were prone to be more vulnerable to SA compared to control cells (Fig. 3G, H and Table S1). BAPTA-AM also synergized with SA and Dox to inhibit cancer cell migration and invasion (Fig. 3I). These results demonstrate that iCa^2+^ signaling is implicated in the expression of IL-6 provoked by genotoxic stress.

iCa^2+^ signaling stimulates PI3K/Akt activation in various circumstances [37–39]. We found that in both untreated and SA-treated cancer cells, iCa^2+^ sequestration not only attenuated Akt phosphorylation at serine (S)473 but also diminished the phosphorylation of S276 on the p65 subunit of the transcription factor NF-κB (nuclear factor-kappa B) (Fig. 3J), whose main function is to transactivate various inflammatory cytokines, including IL-6 [40, 41]. These results imply that iCa^2+^ signaling and IL-6 might be linked via the PI3K/Akt/NF-κB axis. Indeed, genotoxic-mediated IL-6 induction was abolished by inhibiting the PI3K/Akt pathway with either Akt_i_ IV (a potent Akt kinase inhibitor) (Fig. 3K) or LY294002 (a PI3K inhibitor) (Fig. 3K, L). Furthermore, SA-promoted Akt and NF-κB phosphorylations could be diminished by inhibiting the PI3K/Akt pathway (Fig. 3M), indicating that the PI3K/Akt signaling is required for NF-κB activation and IL-6 expression upon genotoxic treatments. In line with this, inhibition of the PI3K/Akt signaling pathway resulted in increased sensitivity of cancer cells to SA (Fig. 3N, O and Table S1) accompanied by attenuating SA-induced Bcl-xL and Mcl-1 gene expressions (Fig. 3P, Q). These results collectively demonstrate that the iCa^2+^/PI3K/Akt/NF-ĸB signaling axis mediates IL-6 expression and plays an essential role in cancer cell tolerance to genotoxic stress.

### Genotoxic stress induces p53-dependent Caspase 2-mediated MDM2 cleavage to promote p53 protein accumulation

**Fig. 4:**
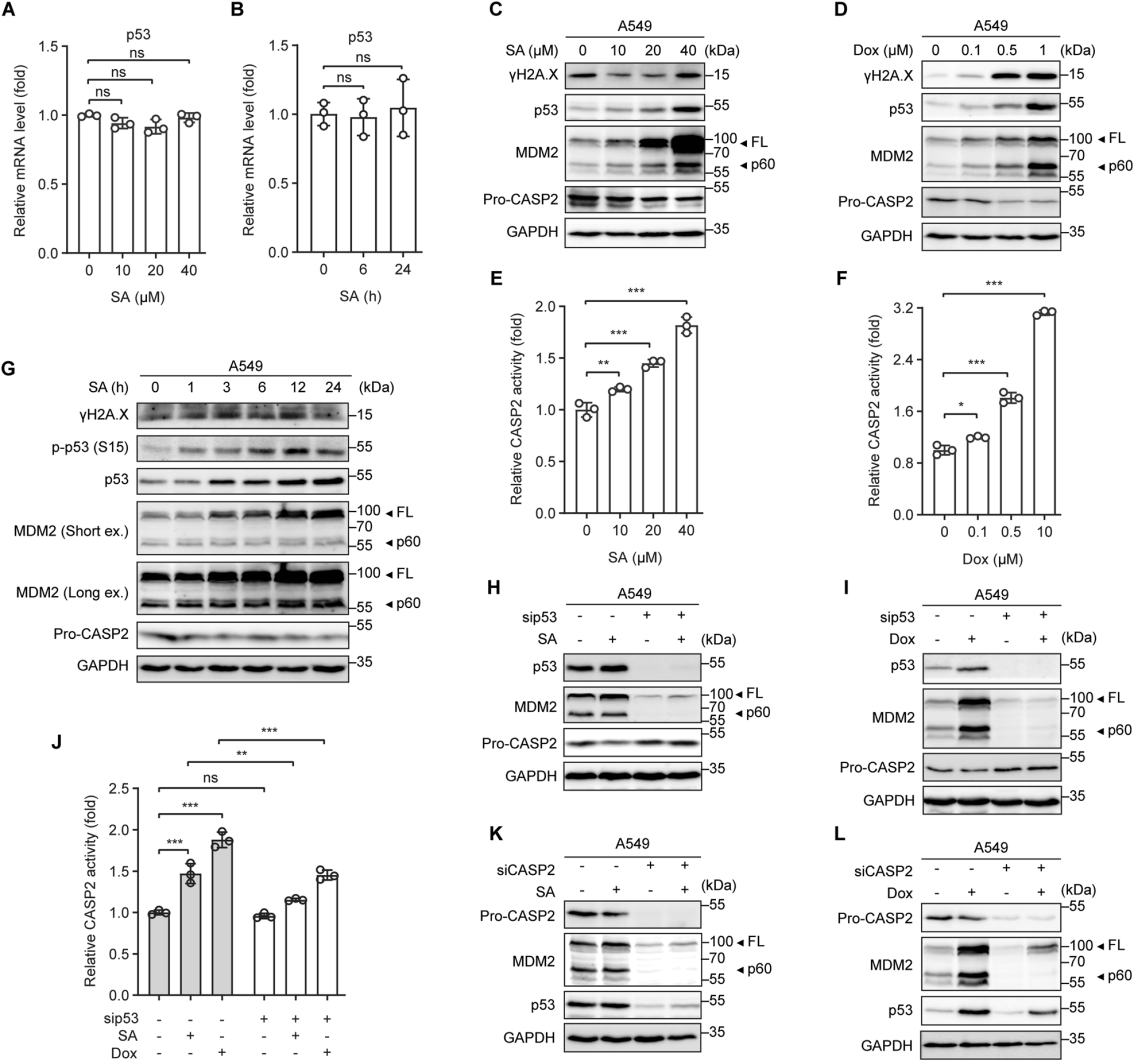
Genotoxic stress induces p53-dependent Caspase 2-mediated MDM2 cleavage to promote p53 protein accumulation. **A**, **B** p53 gene expression in A549 cells treated (**A**) with increasing concentrations of SA for 24 h or (**B**) with 20 µM SA for the indicated time periods was measured by qRT-PCR. **C**, **D** Western blot analysis of γH2A.X, p53, MDM2, and pro-Caspase-2 (Pro-CASP2) protein levels in A549 cells treated with increasing concentrations of (**C**) SA or (**D**) Dox for 24 h. **E**, **F** Caspase-2 (CASP2) activity in A549 cells treated with increasing concentrations of (**E**) SA or (**F**) Dox for 24 h. **G** Western blot analysis of γH2A.X, phosphorylated p53 (S15), total p53, MDM2, and Pro-CASP2 protein levels in A549 cells treated with 20 µM SA for the indicated time periods. **H**, **I** p53, MDM2, and Pro-CASP2 protein levels in control and p53-silenced A549 cells untreated or treated with (**H**) 20 µM SA or (**I**) 0.5 µM Dox for 24 h were determined by western blot analysis. **J** CASP2 activity in control and p53-silenced A549 cells untreated or treated with 20 µM SA or 0.5 µM Dox for 24 h. **K**, **L** p53, MDM2, and Pro-CASP2 protein levels in control and CASP2-silenced A549 cells untreated or treated with (**K**) 20 µM SA or (**L**) 0.5 µM Dox for 24 h were determined by western blot analysis. Error bars represent mean ± SD, *n* = 3. Statistical analysis was performed using unpaired two-tailed Student’s *t* test (**A**, **B**, **E**, **F**) or two-way ANOVA with Tukey’s multiple comparisons test (**J**). *p ≤ 0.05; **p ≤ 0.01; ***p ≤ 0.001; ns, not significant. The full length uncropped original western blots related to this figure are provided in the Supplemental Material file.

After characterizing the Panx1/P2Rs/iCa^2+^/PI3K/Akt/NF-ĸB network as an upstream signaling axis driving IL-6 expression, we established the regulatory link between genotoxic stress and this signaling pathway. Considering our data showing that SA strongly induces IL-6 expression in A549 (p53 WT) but not in H1299 (p53-null) cells (Figs. 1E, F and S1A–C, E), we hypothesized that p53 might be involved in the induction of IL-6 by genotoxic stress. Strikingly, SA had no significant effect on p53 gene expression either in a dose-dependent (Fig. 4A) or time-dependent manner (Fig. 4B). In contrast, p53 protein expression was increased steadily and dose-dependently with either SA or Dox treatment (Fig. 4C, D), indicating that p53 is regulated at the protein level without changes in mRNA expression.

p53 protein expression can be governed by p53-dependent Caspase-2-mediated cleavage of MDM2, a negative regulator of p53 [42, 43]. In agreement with increased p53 protein levels, SA or Dox treatment resulted in Caspase-2 activation, as evidenced by the dose-dependent reductions of its pro-form (Pro-CASP2) (Fig. 4C, D) and increases in its activity towards VDVAD-AFC substrate (Fig. 4E, F), concomitant with elevating MDM2 cleavage products, particularly the p60 fragments (Fig. 4C, D). However, we observed a difference in the protein expression patterns of the DNA double-strand breaks indicator γH2A.X between SA- and Dox-treated cells. γH2A.X protein abundance was dose-dependently induced in Dox-treated (Fig. 4D) but not in SA-treated cells (Fig. 4C) at 24 h post-treatment. In SA-treated cells, γH2A.X was weakly increased at the earliest time point observed (1 h), reached the maximal levels at 3 h, maintained from 6 h until 12 h, and was subsequently reduced at 24 h post-treatment (Fig. 4G). Correspondingly, p53 phosphorylation was induced by SA at 1 h, but peaked at 12 h, followed by a slight decrease at 24 h post-treatment. SA also promoted Caspase-2 activation and MDM2 cleavage as early as 1 h and onward (Fig. 4G). Because p53 is a downstream target of MDM2, p53 protein abundance was thus accumulated at the later time points, from 3 h onward following SA treatment (Fig. 4G). The results suggest that, unlike Dox, which triggers persistent DNA damage even at low concentrations, SA at concentrations as high as 20 µM induces reparable DNA damage at early time points to promote p53 protein accumulation.

Caspase-2 can be activated via p53-dependent or -independent mechanisms [42–44]. In SA- or Dox-treated cells, p53 silencing increased Pro-CASP2 levels (Fig. 4H, I) and attenuated Caspase-2 activity (Fig. 4J), suggesting that Caspase-2 is activated via a p53-dependent mechanism under genotoxic stress. Moreover, Caspase-2 depletion abolished MDM2 cleavage and p53 protein expression under both basal and genotoxic conditions (Fig. 4K, L). Taken together, these results indicate that genotoxic effects, either reversible or irreversible, trigger p53-dependent Caspase-2-mediated MDM2 cleavage to induce p53 protein accumulation.

### p53 transactivates Panx1 to drive IL-6 induction

**Fig. 5:**
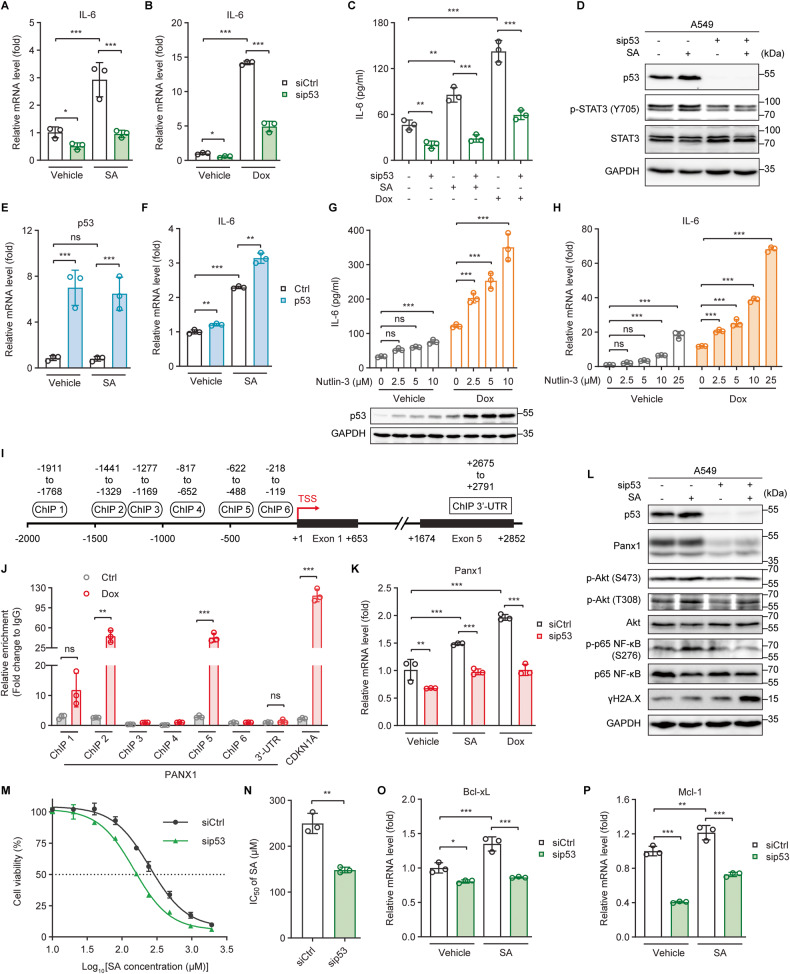
p53 transactivates Panx1 to drive IL-6 induction. **A**, **B** qRT-PCR analysis of IL-6 gene expression in control (siCtrl) and p53-silenced (sip53) A549 cells untreated or treated with (**A**) 20 µM SA or (**B**) 0.5 µM Dox for 24 h. **C** The concentration of IL-6 present in the culture supernatants of siCtrl and sip53 A549 cells untreated or treated with 20 µM SA or 0.5 µM Dox for 24 h was determined by ELISA. **D** Western blot analysis of p53, phosphorylated STAT3 (Y705), and total STAT3 protein levels in siCtrl and sip53 A549 cells untreated or treated with 20 µM SA for 24 h. **E**, **F** qRT-PCR analysis of (**E**) p53 and (**F**) IL-6 gene expression in control (Ctrl) and p53-overexpressing (p53) A549 cells untreated or treated with 20 µM SA for 24 h. **G** The concentration of IL-6 present in the culture supernatants (upper panel) or the p53 protein levels (lower panel) of A549 cells untreated or treated with 0.5 µM Dox in the presence of increasing concentrations of the MDM2 inhibitor Nutlin-3 for 24 h was determined by ELISA or western blot, respectively. **H** qRT-PCR analysis of IL-6 gene expression in A549 cells untreated or treated with 0.5 µM Dox in the presence of increasing concentrations of Nutlin-3 for 24 h. **I** Schematic diagram of the human PANX1 gene locus with six potential p53 binding regions, −1911 to −1768 (ChIP 1), −1441 to −1329 (ChIP 2), −1277 to −1169 (ChIP 3), −817 to −652 (ChIP 4), −622 to −488 (ChIP 5), and −218 to −119 (ChIP 6), on the PANX1 promoter (upstream of TSS) and a 3′-untranslated region (ChIP 3′-UTR, +2675 to +2791, downstream of TSS), identified with the JASPAR program [46]. TSS, transcription start site. **J** Chromatin immunoprecipitation and quantitative real-time PCR (ChIP-qPCR) analysis of the relative enrichment of p53 at the indicated PANX1 promoter regions in A549 cells with or without 0.5 µM Dox treatment for 24 h. The relative enrichments of p53 at the CDKN1A (p21) promoter region (−2292 to −2169, upstream of TSS) and the 3′-untranslated (3′-UTR) region of the PANX1 gene were measured as positive and negative controls, respectively. **K** Panx1 gene expression in siCtrl and sip53 A549 cells untreated or treated with 20 µM SA or 0.5 µM Dox for 24 h was measured by qRT-PCR. **L** Western blot analysis of p53, Panx1, phosphorylated Akt (S473 and T308), total Akt, phosphorylated p65 NF-ĸB (S276), total p65 NF-ĸB, and γH2A.X protein levels in siCtrl and sip53 A549 cells untreated or treated with 20 µM SA for 24 h. **M** Dose-response curves showing the survival of siCtrl and sip53 A549 cells in response to increasing concentrations of SA treatment for 24 h. **N** The IC_50_ values of SA against siCtrl and sip53 A549 cells were calculated from the nonlinear regression curves in Fig. 5M. Cell viability was measured by MTT assays. **O**, **P** The mRNA levels of the anti-apoptotic genes (**O**) Bcl-xL and (**P**) Mcl-1 in siCtrl and sip53 A549 cells untreated or treated with 20 µM SA for 24 h were measured by qRT-PCR. Error bars represent mean ± SD, *n* = 3. Statistical analysis was performed using two-way ANOVA with Tukey’s multiple comparisons test (**A**–**C**, **E**–**H**, **K**, **O**, **P**) or unpaired two-tailed Student’s *t* test (**J**, **N**). *p ≤ 0.05; **p ≤ 0.01; ***p ≤ 0.001; ns not significant. The full length uncropped original western blots related to this figure are provided in the Supplemental Material file.

Next, we examined the role of p53 in genotoxic stress-mediated IL-6 expression. Knockdown of p53 expression diminished both basal and genotoxic-induced IL-6 gene expressions in A549 (Fig. 5A, B), MCF-7 (Fig. S4A, B), and Hela (Fig. S4C, D) cells that express WT p53. p53 silencing also mitigated IL-6 protein levels in the culture supernatants of both control and SA/Dox-treated A549 cells (Fig. 5C) and inhibited both basal and SA-induced STAT3 phosphorylations (Fig. 5D). Conversely, expression of exogenous WT p53 led to a roughly 1.4-fold increase in SA-induced IL-6 expression (Fig. 5E, F). Similarly, enhanced stabilization of p53 with Nutlin-3, a small-molecule MDM2 inhibitor, further advanced both Dox-induced IL-6 protein production and gene expression in a dose-dependent manner (Fig. 5G, H). These findings demonstrate clearly that p53 plays an essential role in genotoxic-mediated IL-6 induction.

p53 functions primarily as a transcription factor that recognizes target genes by binding to p53 consensus response elements (p53REs) located within the promoter regions [45]. Using the JASPAR program [46], we identified six potential binding sites for p53 within the promoter regions (ChIP 1–6) of the PANX1 gene locus (Fig. 5I). Our chromatin immunoprecipitation (ChIP) assay coupled with quantitative real-time PCR (qRT-PCR) analysis revealed that p53 was significantly enriched at the −1441 to −1329 (ChIP 2) and the −622 to −488 (ChIP 5) regions, but not at the other four regions (ChIP 1, 3, 4, and 6), upon Dox treatment (Fig. 5J). Furthermore, p53 knockdown reduced Panx1 gene expressions and protein levels in both untreated and SA-treated cells (Fig. 5K, L). We further investigated the role of p53 in the Panx1 downstream PI3K/Akt/NF-ĸB signaling axis. p53 depletion diminished the phosphorylations of S473 and threonine (T)308 on Akt and S276 on the p65 subunit of NF-κB together with aggravating γH2A.X protein levels under both basal and SA treatment conditions (Fig. 5L). Consistently, p53 knockdown strengthened the cytotoxic effects of SA against A549 cells (Fig. 5M, N and Table S1). The gene expressions of Bcl-xL and Mcl-1 were also diminished upon p53 loss in both untreated and SA-treated cells (Fig. 5O, P). Taken together, the results suggest that p53 directly transactivates Panx1 to induce IL-6 expression under genotoxic conditions and thereby linking it to the genotoxic fitness and adaptability of cancer cells.

### IL-6 exhibits pro-survival and -metastatic effects by promoting alternative (M2-like) polarization of macrophages

**Fig. 6:**
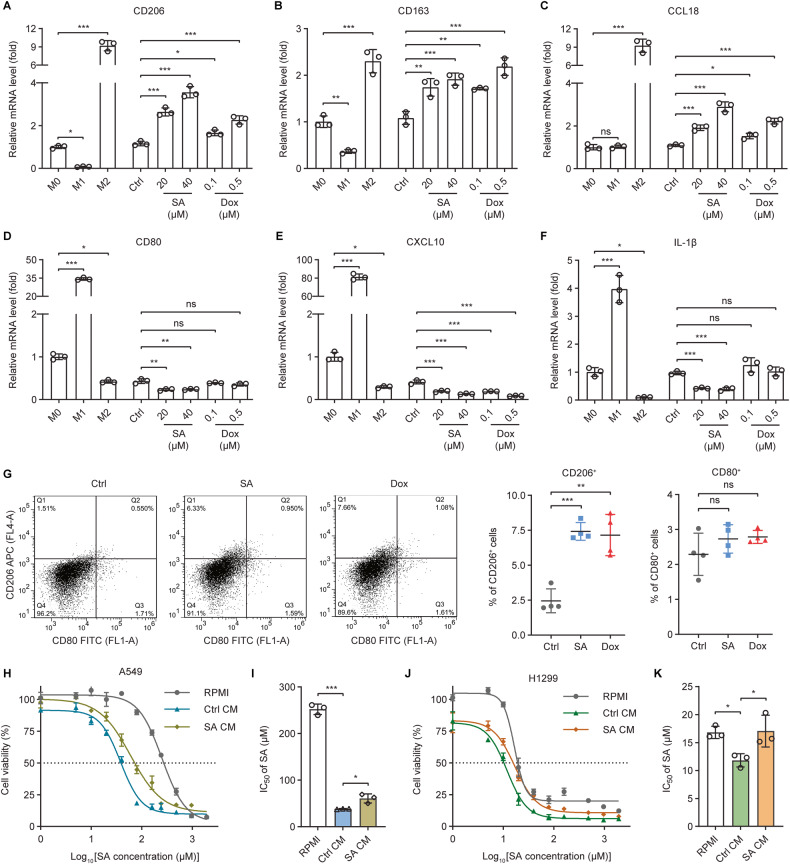
Conditioned medium from genotoxic-exposed cancer cells stimulates alternative (M2-like) polarization of macrophages. **A**–**F** The mRNA levels of (**A**–**C**) M2- (CD206, CD163, and CCL18) and (**D**–**F**) M1-associated (CD80, CXCL10, and IL-1β) macrophage markers were analyzed in THP-1-derived M0 and positive control M1/M2-polarized macrophages as well as in macrophages stimulated with conditioned medium from control (Ctrl), SA-treated (20 or 40 µM), or Dox-treated (0.1 or 0.5 µM) A549 cells using qRT-PCR. **G** Flow cytometric analysis of cell surface expressions of the M1- (CD80) and M2-associated (CD206) macrophage markers in macrophages stimulated with conditioned medium from control (Ctrl), SA-treated (20 µM), or Dox-treated (0.1 µM) A549 cells. Cells were double-stained with FITC-labeled anti-CD80 and APC-labeled anti-CD206 antibodies. **H**, **J** Dose-response curves showing the survival of (**H**) A549 or (**J**) H1299 cells cultured in RPMI medium (RPMI) or the conditioned medium, Ctrl CM or SA CM, from macrophages stimulated with the conditioned medium of control or 20 µM SA-treated A549 cells, respectively, in response to increasing concentrations of SA treatment for 24 h. **I**, **K** The IC_50_ values of SA against (**I**) A549 or (**K**) H1299 cells cultured in RPMI, Ctrl CM, or SA CM were calculated from the nonlinear regression curves in Fig. 6H or J, respectively. Cell viability was measured by MTT assays. Error bars represent mean ± SD, *n* = 3. Statistical analysis was performed using one-way ANOVA with Tukey’s multiple comparisons test. *p ≤ 0.05; **p ≤ 0.01; ***p ≤ 0.001; ns not significant.

The responses of cancer cells to genotoxic therapeutics are influenced not only by cell-intrinsic signaling pathways but also by cell-extrinsic mechanisms [5]. Previous studies have highlighted that alternatively activated (M2-polarized) macrophages are associated with accelerated tumor aggressiveness, metastasis, and treatment failure [47–49]. We next sought to understand whether genotoxic treatments of cancer cells affect macrophage phenotype polarization to modulate cancer cell responsiveness. Human monocytic THP-1 cells were primed with phorbol-12-myristate-13-acetate (PMA) to induce differentiation into nonpolarized (M0) macrophages (Fig. S5A–G). Strikingly, stimulating M0 macrophages with the conditioned medium (CM) collected from SA or Dox-treated A549 cells (Fig. S5A) augmented the gene expressions of the M2-associated markers CD206, CD163, and CCL18, but not the M1-associated markers CD80, CXCL10, and IL-1β (Fig. 6A–F). These observations were not made in macrophages stimulated with the CM from control A549 cells. Moreover, flow cytometric analysis showed significant increases in the percentage of CD206^+^, but not CD80^+^ macrophages, when they were stimulated with the CM from SA/Dox-treated cancer cells as compared to that of the macrophages stimulated with the CM from control cancer cells (Fig. 6G). Macrophage-conditioned media, referred to as Ctrl CM and SA CM, were then collected from macrophages stimulated with the conditioned media derived from Ctrl and SA-treated A549 cells, respectively (Fig. S5A). Significantly, SA CM exhibited a reduced capacity to synergize with SA in killing cancer cells as compared to Ctrl CM (Fig. 6H–K and Table S1). These results suggest that factors secreted by cancer cells under genotoxic conditions promote the M2-like polarization of macrophages that exhibits diminished anticancer effects.

**Fig. 7:**
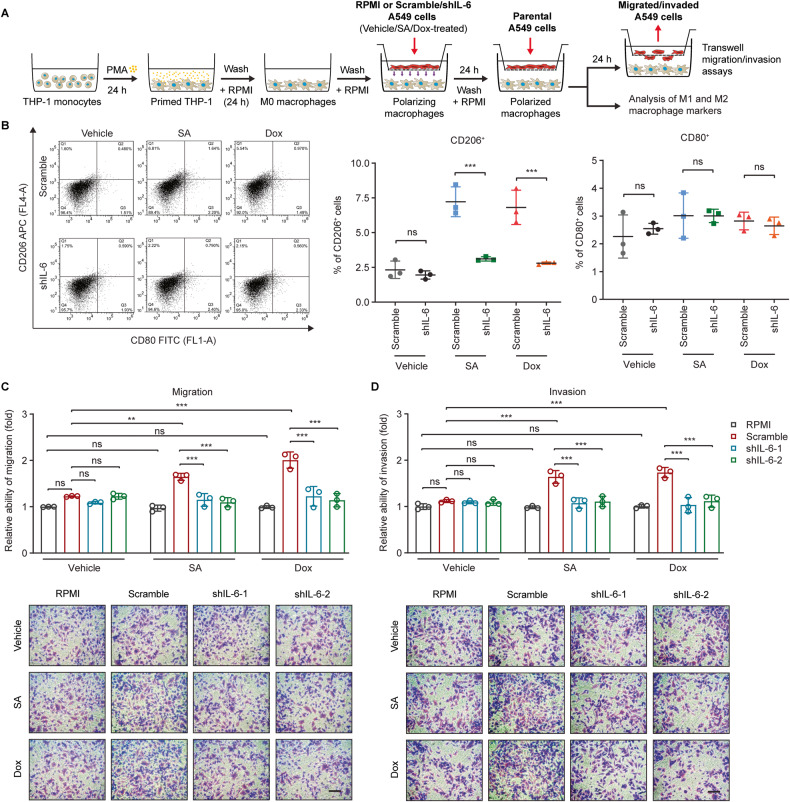
IL-6 secreted by cancer cells plays a critical role in M2-like macrophage polarization. **A** THP-1 monocytes were differentiated into M0 macrophages, co-cultured with control or IL-6-silenced A549 cells with and without SA/Dox treatment to induce macrophage polarization, and co-cultured with parental A549 cells to stimulate cancer cell migration and invasion as depicted. **B** Flow cytometric analysis of cell surface expression of the M1- (CD80) and M2-associated (CD206) macrophage markers in macrophages co-cultured with control (scramble) or IL-6-silenced (shIL-6) A549 cells with and without 20 µM SA or 0.1 µM Dox treatment for 24 h. Cells were double-stained with FITC-labeled anti-CD80 and APC-labeled anti-CD206 antibodies. **C**, **D** The (**C**) migratory and (**D**) invasive abilities of A549 cells co-cultured with macrophages stimulated with RPMI, control (scramble), or IL-6-silenced (shIL-6-1 and shIL-6-2) A549 cells with and without 20 µM SA or 0.1 µM Dox treatment for 24 h were measured with transwell assays. Scale bar: 100 µm. Error bars represent mean ± SD, *n* = 3. Statistical analysis was performed using two-way ANOVA with Tukey’s multiple comparisons test. *p ≤ 0.05; **p ≤ 0.01; ***p ≤ 0.001; ns not significant.

IL-6 skews macrophages toward an M2-like phenotype under various circumstances [13, 14]. We hypothesized that IL-6 secreted by genotoxic treatments of cancer cells plays a key role in driving M2 macrophage polarization. To this end, M0 macrophages were co-cultured with the control (scramble) or IL-6-silenced (shIL-6-1 and shIL-6-2) A549 cells with and without SA or Dox treatment (Fig. 7A). Interestingly, IL-6 knockdown in the stimulating SA/Dox-treated cancer cells diminished the expressions of the M2-associated markers CD206 and CD163 in their co-cultured macrophages, whereas this effect was not observed for the M1-associated marker CD80 (Figs. S5H–J and 7B). Furthermore, macrophages co-cultured with SA/Dox-treated IL-6-silenced A549 cells exhibited decreased pro-migratory and -invasive effects on cancer cells compared to those co-cultured with SA/Dox-treated control cells (Fig. 7C, D). Altogether, these data imply that IL-6 secreted by cancer cells undergoing genotoxic stress enables macrophages to gain an M2-like phenotype that possesses diminished anti-survival activities but augmented pro-migratory and invasive potentials.

## Discussion

The function of WT p53 as a tumor suppressor has primarily been ascribed to its capacity to transcriptionally regulate the expression of downstream target genes involved in proliferation inhibition and programmed cell death in response to a wide range of cellular stresses, including DNA damage [16, 18]. Here, we report that genotoxic stress triggers DNA damage and p53 accumulation (Fig. 4C, D, G) together with hampering the viability (Figs. 1H–K and S1L, M) and metastatic potentials of cancer cells (Fig. 1O, P). Intriguingly, while the anti-migratory and invasive activities of p53 induced upon treatment with the genotoxic agent SA have been clearly demonstrated in our previous study [19], p53 appears not to play an essential role in SA-mediated cell death, as loss of p53 enhances, but not mitigates, the cytotoxic effects of SA on cancer cells (Fig. 5M, N). Thus, it is likely that p53 does not always function as a tumor suppressor in cancer cells experiencing DNA damage. We further discover the detailed molecular mechanism underlying the potential oncogenic role of p53 under both reversible and irreversible genotoxic conditions. p53 stimulates the induction of the pro-survival and -metastatic cytokine IL-6 by transactivating the major ATP release channel Panx1 to hamper the responsiveness of cancer cells to genotoxic treatments. Our findings affirm the report that genotoxic stress activates p53 and leads to the production of the IL-6 family (JAK/STAT) cytokines *Upd*, *Upd2*, and *Upd3* in *Drosophila* oncogenic Ras tissues [50].

The expression levels and activities of p53 are regulated via multiple mechanisms [17]. p53 protein expression can be induced by Caspase-2-mediated cleavage of its negative regulator MDM2 in response to DNA damage [42, 43]. Notably, DNA damage stimulates the production of interferons (IFNs), including IFN-α, -β, and -λ, and activates IFN signaling [51, 52], which is also a crucial mediator of p53 transcription [53] and p53-dependent tumor-suppressive activities [53, 54]. We show here that genotoxic-induced DNA damage amplifies the protein accumulation but not the mRNA levels of p53 (Fig. 4A–D). These observations corroborate the notion that p53 is controlled at the protein level via Caspase-2-mediated MDM2 cleavage under genotoxic stress and suggest that the signaling pathways that regulate p53 transcription, such as the IFN signaling pathway, are not implicated in the DNA damage-induced p53 expression.

IL-6 is a pleiotropic cytokine with diverse biological activities, most notably related to tumor-promoting processes [7, 55]. These effects of IL-6 are attributed to the activation of two major pro-tumorigenic and -metastatic signaling pathways, including the JAK/STAT3 (Janus kinase/signal transducer and activator of transcription 3) and the JAK/SHP2/MAPK (SH2 domain-containing protein tyrosine phosphatase 2/mitogen‑activated protein kinase) pathways [6], which play critical roles in DNA damage repair, anti-apoptotic, and pro-metastatic processes [56, 57]. Consistently, we show here that genotoxic stress-produced IL-6 stimulates STAT3 phosphorylation (Fig. 1G, L) together with mitigating DNA damage (Fig. 1L), enhancing the expression of anti-apoptotic proteins (Fig. 1M, N), and promoting genotoxic tolerance (Figs. 1H–K and S1L, M). IL-6 also maintains the migratory and invasive abilities of cancer cells to hamper the anti-metastatic activities of genotoxic agents (Fig. 1O, P). Furthermore, previous studies demonstrate that exogenous sources of IL-6 reduce p53 stabilization and protein expression, either via STAT3-dependent or -independent mechanism, leading to increased cancer cell survival under genotoxic conditions [10, 58]. Accordingly, it is reasonable to hypothesize that abolishing IL-6 or targeting its associated signaling may reinforce the tumor-suppressive activities of WT p53, thereby enhancing cancer cell responsiveness to genotoxic anticancer drugs.

IL-6 expression and activity are regulated by various transcriptional and posttranscriptional mechanisms in a context-dependent fashion [59]. Remarkably, NF-ĸB is activated by DNA-damaging agents [60] and mediates IL-6 gene expression under genotoxic conditions [41]. In line with this, we observe an increase in NF-ĸB activity together with an upregulation of IL-6 expression upon genotoxic treatments. Furthermore, we show that genotoxic stress-activated NF-ĸB and -induced IL-6 expression are controlled by the Panx1/P2Rs-directed iCa^2+^/PI3K/Akt signaling pathway as the NF-ĸB activity and IL-6 gene expression provoked by genotoxic treatments are dramatically attenuated following pharmacological inhibition of the PI3K/Akt activity or sequestration of cytosolic Ca^2+^. Our observation is supported by multiple studies illustrating the involvement of Akt [61, 62], iCa^2+^ signaling [25, 63], P2Rs [64, 65], extracellular ATP [64, 66], and Panx1 [25] in the activation of NF-ĸB.

Panx1 is the most widely expressed and extensively studied among three members (Panx1, Panx2, and Panx3) of the pannexin family [67, 68]. It is one of the major conduits for ATP release and is implicated in tumor growth, metastasis, and anticancer drug resistance [67]. However, the underlying mechanisms for Panx1 transcriptional regulation and for the pro-survival and -metastatic functions of Panx1 remain poorly understood. We now suggest that Panx1 is directly transactivated by p53 (Fig. 5J) and exhibits its tumor-supporting effects, at least in part, by mediating ATP release and subsequent activation of the P2Rs/iCa^2+^/PI3K/Akt/NF-ĸB signaling axis to drive IL-6 expression under genotoxic stress. Panx1 gene expression and protein levels are controlled by p53 under both basal and genotoxic conditions (Fig. 5K, L). Consistently, p53 silencing significantly diminishes the basal and genotoxic-induced Akt and NF-ĸB activation (Fig. 5L) and IL-6 expression (Fig. 5A–C). These results are in agreement with the observations seen upon sequestration of iCa^2+^ (Fig. 3E, F, J). On the contrary, the basal IL-6 expression is unaffected following the inhibition of the Panx1 (Fig. 2G, H) or P2Rs (Fig. 3C, D) activity. Furthermore, the depletion of p53, the chelation of iCa^2+^, or the suppression of the PI3K/Akt signaling pathway appears to have a more remarkable impact on genotoxic-induced IL-6 expression than the inhibition of the Panx1 or P2Rs activity. Our findings suggest that p53 regulates the iCa^2+^/PI3K/Akt/NF-ĸB signaling activation and IL-6 expression via Panx1/P2Rs under genotoxic stress but not basal circumstances and that there may be other mechanisms independent of Panx1 and P2Rs underlying the regulation of the iCa^2+^/PI3K/Akt/NF-ĸB/IL-6 axis by p53 under genotoxic conditions. For example, p53 represses the expression of the transient receptor potential channel melastatin 4 (TRPM4) to potentiate extracellular Ca^2+^ influx [69]. p53 directly binds to the nuclear Akt [70] or NF-ĸB [71] to enable the activation of these factors. Remarkably, p53 mediates NF-ĸB-induced expressions of genes related to innate immune response, including IL-6, by regulating the activation of the DNA sensing adapter STING (stimulator of interferon genes) in response to genotoxic treatments [72].

Cancer cells gain chemoresistance properties via both cell-intrinsic signaling pathways and cell-extrinsic factors, such as cytokines and growth factors secreted in the tumor microenvironment [5]. Our findings suggest that IL-6 serves as a cell-extrinsic factor secreted by cancer cells in response to genotoxic treatments (Figs. 1E, F and S1E) to counteract the anti-survival and -metastatic activities of these anticancer agents. In addition to a direct autocrine effect on cancer cells themselves, IL-6 acts in a paracrine manner to skew macrophage polarization into an M2-like phenotype (Figs. 7B and S5H–J). The promoting effects of IL-6 on M2-like macrophage polarization and its underlying molecular mechanisms have been reported under various conditions [13, 14, 73], however, these effects have never been previously explored under genotoxic stress. Our data demonstrate that genotoxic-induced IL-6-mediated M2-like macrophage polarization not only dampens their anti-survival activities (Fig. 6H–K) but also augments their pro-migratory and -invasive effects on cancer cells (Fig. 7C, D). These observations are in line with previous studies illustrating that M2 macrophages can secrete various cytokines and factors into the tumor microenvironment to promote cancer cell growth, metastasis, and treatment resistance [47–49, 74]. Accordingly, combining IL-6 blockades with genotoxic agents not only impairs the abilities of cancer cells to evade genotoxic effects but may also alleviate the induction of the pro-tumorigenic M2-like macrophages in the tumor microenvironment under genotoxic conditions, thereby contributing to improving treatment outcomes.

**Fig. 8:**
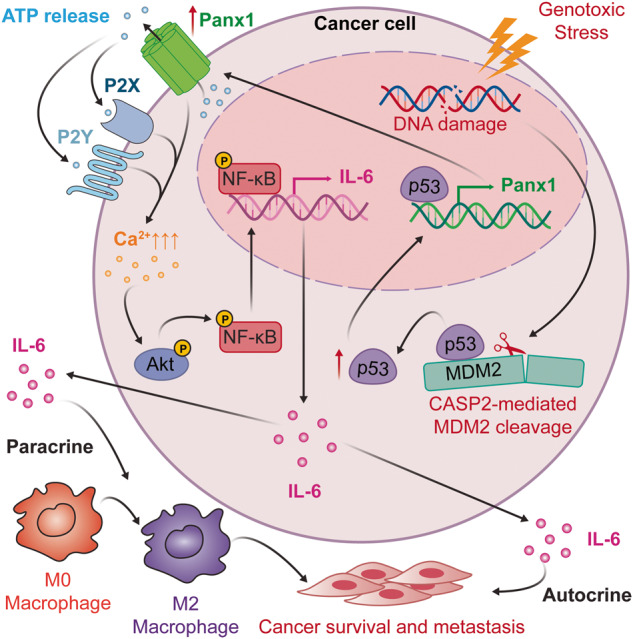
A putative schematic presentation of tumor suppressor p53-mediated IL-6 expression under genotoxic stress. Wild-type p53 transcriptionally controls the major ATP release channel Panx1 that mediates IL-6 induction via the P2Rs/iCa^2+^/PI3K/Akt/NF-κB signaling axis. IL-6 mitigates cancer cell sensitivity to genotoxic treatments and maintains the metastatic abilities of cancer cells via both autocrine (cell response to its own secreted IL-6) and paracrine (action of IL-6 secreted by cancer cells to promote M2-like macrophage polarization) mechanisms.

In summary, our study illuminates a molecular explanation for genotoxic stress-induced IL-6 via p53-directed Panx1 expression (Fig. 8). The results have significant implications for improving the efficacy of genotoxic anticancer therapies. Currently, multiple strategies have been used to restore the tumor-suppressive activities of WT p53 in p53-compromised cancers to enhance cancer cell responses to genotoxic chemotherapies [75, 76]. In light of our findings, expression of WT p53 may stimulate IL-6 induction that promotes cancer cell fitness and tolerance to genotoxic anticancer agents, thereby hampering the effectiveness of those anticancer therapeutics. Therefore, IL-6 blockade can work synergically with genotoxic agents to effectively suppress p53 WT cancer growth or can be used in combination with p53 restoration therapy to improve the effectiveness of genotoxic anticancer chemotherapies in p53-compromised cancers.

## Materials and methods

### Cell cultures, treatments, and reagents

A549, H1299, MCF-7, and Hela cells were purchased from the American Type Culture Collection (ATCC, Manassas, VA, USA). The human monocytic THP-1 cell line was kindly provided by Dr. Yu-Ting Chou (Institute of Biotechnology, College of Life Sciences and Medicine, National Tsing Hua University, Taiwan). A549, H1299, and THP-1 cell lines were grown in Roswell Park Memorial Institute (RPMI) 1640 medium supplemented with 10% heat-inactivated fetal bovine serum (FBS), 0.22% sodium bicarbonate, 2 mM l-glutamine, and 100 units/ml penicillin/streptomycin. MCF-7 and Hela cell lines were cultured in Dulbecco’s modified Eagle’s medium (DMEM) containing 10% FBS, 0.37% sodium bicarbonate, 2 mM l-glutamine, and 100 units/ml penicillin/streptomycin. All cell lines were maintained in a humidified incubator at 37 °C and 5% CO_2_. All reagents for cell cultures were purchased from Invitrogen Gibco (Grand Island, NY, USA). Recombinant human IL-6, IL-4, and IFN-γ proteins were purchased from PeproTech (Cranbury, NJ, USA). Other chemicals in this study were obtained from Sigma-Aldrich (St. Louis, MO, USA) unless specified.

Sodium arsenite (SA) (Merck, Darmstadt, Germany) and doxorubicin (Dox) (Sigma-Aldrich) were dissolved in sterile double-distilled water (ddH_2_O) while cisplatin (CisPt) (Sigma-Aldrich) was dissolved in dimethyl sulfoxide (DMSO) (Sigma-Aldrich) and added to the culture media from concentrated stocks. The same amount of solvents (ddH_2_O or DMSO) was added to the control wells. Carbenoxolone disodium salt (CBX) (Sigma-Aldrich) and Suramin sodium salt (Suramin) (Merck Darmstadt, Germany) were dissolved in ddH_2_O to create 10 and 20 mM stock solutions, respectively. BAPTA-AM (Enzo Life Sciences, Farmingdale, NY, USA), Akt_i_ IV (Calbiochem, San Diego, CA, USA), LY294002 (Calbiochem), and Nutlin-3 (Selleck Chemicals, Houston, TX, USA) were dissolved in DMSO to create 10 mM stock solutions. A 1-h pre-treatment with CBX (50 μM), Suramin (50 μM), BAPTA-AM (10 μM), Akt_i_ IV (10 μM), or LY294002 (10 μM), or a 6-h pre-treatment with Nutlin-3 (2.5, 5, 10, or 25 μM) or recombinant human IL-6 (50 or 20 ng/ml) was done prior to SA, Dox, or CisPt addition. ddH_2_O was used as the control solvent for the CBX, the Suramin, and the IL-6 treatments, while DMSO was used as the control solvent for the BAPTA-AM, the Akt_i_ IV, the LY294002, and the Nutlin-3 treatments.

### Total RNA extraction, reverse transcription, and quantitative real-time PCR

Total RNA was isolated using TRIzol reagent (Invitrogen, Carlsbad, CA, USA) according to the manufacturer’s instructions. Three μg of the extracted RNA was reverse-transcribed into complementary (c)DNA in a final volume of 20 μl with a RevertAid First Strand cDNA Synthesis Kit (Invitrogen, Carlsbad, CA, USA). The resulting cDNA was used for performing qRT-PCR using SYBR Green PCR Master Mix (Applied Biosystems, Foster City, CA, USA) on a StepOnePlus Real-Time PCR system (Applied Biosystems, Foster City, CA, USA). Data were acquired and analyzed using StepOne Software v2.3 (Thermo Fisher Scientific, Leicestershire, UK). Glyceraldehyde-3-phosphate dehydrogenase (GAPDH) was used as a reference gene for A549, H1299, MCF-7, and Hela cells, and ubiquitin C (UBC) was used as a reference gene for THP-1 cells. The expression of target genes was measured and compared on the basis of equivalent GAPDH or UBC transcripts using the delta-delta Ct (also known as the 2^−ΔΔCt^) method. Primers used in qRT-PCR were purchased from Integrated DNA Technologies (Coralville, IA, USA). Information on primer sequences used in this study is provided in Table S2.

### Enzyme-linked immunosorbent assay

Cells were seeded in 24-well plates (Corning Inc., Durham, NC, USA; 3 × 10^4^ cells per well) and treated with the appropriate reagents. The cultured media were collected after 24 h of treatments and centrifuged at 1000 × *g* for 5 min at 4 °C to remove cell debris. IL-6 concentration in the cultured media was measured using the Human IL-6 Uncoated ELISA Kit (Invitrogen, Carlsbad, CA, USA) according to the manufacturer’s protocol.

### Lentivirus knockdown system

The human IL-6 shRNA constructs, pLKO.1-shIL-6-1 (TRCN0000059207) and pLKO.1-shIL-6-2 (TRCN0000059206), and the pLKO.1-scramble construct (ASN0000000004) were obtained from the National RNAi Core Facility, Academia Sinica (Taipei, Taiwan). HEK293T cells were co-transfected with 12 μg of the scramble or IL-6 shRNA vectors cloned in the pLKO.1 plasmid, 9 μg of the psPAX2 packaging plasmid, and 3.6 μg of the pMD2.G envelope plasmid using Maestrofectin transfection reagent (MaestroGen, Hsinchu City, Taiwan) according to the manufacturer’s instructions. The lentiviral particles were collected from the medium of HEK293T cells at 72 h post-infection by centrifugation at 300 × *g* for 5 min. The supernatant was then collected and subsequently filtered through a 0.45 μm pore size membrane before being stored at −80 °C or used immediately to infect A549 cells. For lentiviral infection, A549 cells were seeded at 1 × 10^5^ cells per well in six-well plates (Corning Inc., Wujiang, Jiangsu, China) overnight, and then 1 ml of viral supernatant was added to cells in the presence of 10 μg Polybrene (Sigma-Aldrich) for 4 h in a humidified incubator at 37 °C and 5% CO_2_. Following the viral infection, the medium of infected cells was replaced by complete RPMI medium, and cells were incubated for an additional 44 h. Subsequently, the cells were subjected to selection in culture media containing 1.5 μg/ml puromycin [77] (Gibco) until no non-transduced cells were alive. Detailed information on the shRNA sequences is provided in Table S3.

### Cell viability assay

Cells (8000 cells per well) were seeded in 96-well cell culture plates (TPP Techno Plastic Products AG, Trasadingen, Switzerland) overnight to allow attachment before being treated with the appropriate reagents for 24 h. Cell viability was evaluated using the colorimetric MTT assay (Alfa Aesar, Thermo Fisher Scientific, Leicestershire, UK) following the manufacturer’s instructions. Briefly, cells were incubated in culture media containing 0.4 mg/ml of MTT reagent for 2.5 h in a humidified incubator at 37 °C and 5% CO_2_. The medium was then carefully removed and the formazan crystals were dissolved in DMSO (Sigma-Aldrich) at 37 °C for 40 min. The absorbance was measured at 550 nm against a reference wavelength of 650 nm using a microtiter plate reader (Bio-Rad Hercules, CA, USA). The half maximal inhibitory concentration (IC_50_) values were calculated from the nonlinear regression curves of cell survival constructed by plotting the percentage of cell viability versus the logarithm of drug concentrations using GraphPad Prism 9 software (GraphPad Inc., San Diego, CA, USA).

### Western blotting

Cells treated with the indicated reagents for 24 h were washed twice with ice-cold phosphate-buffered saline (PBS) and harvested by gently scraping the cells in ice-cold PBS from cell culture dishes with a cell scraper. Cells were then transferred to centrifuge tubes and subjected to centrifugation at 700 × *g* for 5 min at 4 °C and lysed with ice-cold radioimmunoprecipitation assay (RIPA) lysis buffer (50 mM Tris-HCl (pH 8.0), 150 mM NaCl, 5 mM EDTA (pH 8.0), 1% NP-40, 0.1% SDS, and 0.5% sodium deoxycholate) containing complete protease and phosphatase inhibitor cocktails (Fivephoton Biochemicals, San Diego, CA, USA). Cell lysates were incubated on ice for 40 min with vigorous vortexing every 10 min, followed by centrifugation at 16,000 × *g* for 20 min at 4 °C. The protein concentrations of cell lysate supernatants were determined using the Bio-Rad protein assay (Bio-Rad, Hercules, CA, USA). Proteins in cell lysates were subjected to SDS-PAGE and transferred electrophoretically onto methanol-activated polyvinylidene difluoride (PVDF) membranes (GE Healthcare, Milwaukee, WI, USA) using a transfer cell (Bio-Rad, Hercules, CA, USA). Subsequently, the membranes were blocked with 5% non-fat dry milk in Tris-buffered saline with Tween-20 (TBST) buffer (150 mM NaCl, 10 mM Tris-HCl (pH 8.0), and 0.1% Tween-20) for 1 h before being hybridized at 4 °C overnight with appropriate primary antibodies diluted in 5% bovine serum albumin (BSA)-containing TBST buffer. Antibodies against γH2A.X (S139) (9718), Akt (9272), phospho-Akt (S473) (9271), phospho-Akt (T308) (13038), phospho-p53 (S15) (9286), Panx1 (91137), and phospho-STAT3 (Y709) (9145) were purchase from Cell Signaling Technology (Beverly, MA, USA). Antibodies against p65 NF-ĸB (sc-8008) and phospho-p65 NF-ĸB (S276) (ab194726) were purchased from Santa Cruz Biotechnology (Dallas, TX, USA) and Abcam (Cambridge, UK), respectively. Antibodies against p53 (GTX70214), IL-6 (GTX110527), STAT3 (GTX15523), and GAPDH (GTX100118) were obtained from GeneTex (Hsinchu, Taiwan). Antibodies against Caspase-2 (MAB3507) and MDM2 (MABE340) were from EMD Millipore Corporation (Burlington, NC, USA). All antibodies were used at a 1:1000 dilution except anti-GAPDH (1:10,000 dilution).

After being washed three times for 15 min each with TBST buffer, the membranes were probed for 1 h with the respective horseradish peroxidase (HRP)-conjugated secondary antibodies diluted to 10,000 folds to detect GAPDH or 5000 folds to detect other proteins in 5% non-fat dry milk in TBST buffer followed by being washed three times for 15 min each with TBST buffer. HRP-conjugated goat anti-rabbit IgG (GTX213110-01), sheep anti-mouse IgG (NA931V), and rabbit anti-rat IgG (ab6734) were purchased from GeneTex (Hsinchu, Taiwan), Amersham (GE Healthcare, Buckinghamshire, UK), and Abcam (Cambridge, UK), respectively. Signals were detected by chemiluminescence using a T-Pro LumiLong Plus Chemiluminescent Substrate Kit (M) (T-Pro Biotechnology, New Taipei County, Taiwan) and scanned with an ImageQuant LAS 4000 mini biomolecular imager (GE Healthcare, Milwaukee, WI, USA). Band intensities were quantified with the UN-SCAN-IT gel analysis software (version 6.1) (Silk Scientific, Orem, UT, USA).

### RNA interference

Small interference (si)RNA-mediated gene knockdown experiments were performed using Lipofectamine RNAimax (Invitrogen, Carlsbad, CA, USA) following the manufacturer’s reverse transfection protocol. Cells were transfected with 10 nM Stealth siRNA (Invitrogen) against human p53 or Caspase-2, or 5 nM Silencer Select siRNA (Invitrogen) against IL-6 for 24 h. After transfection, the media of the transfected cells were replaced with complete media, and the cells were incubated for an additional 24 h before receiving appropriate treatments. Stealth RNAi siRNA Negative Control (Invitrogen) and Silencer Select Negative Control No. 1 siRNA (Invitrogen) were used as the controls for the transfections using Stealth and Silencer Select siRNAs, respectively. Detailed information on the siRNA target sequences is provided in Table S4.

### Plasmid construction and transfection

Plasmid DNA for the overexpression of WT p53 was constructed by inserting a WT p53 gene (393 amino acids) into a pcDNA3 plasmid and transfected into A549 cells using Lipofectamine 2000 reagent (Invitrogen) according to the manufacturer’s guideline. After 6 h of transfection, the media of transfected cells were replaced by complete media, and cells were incubated for an additional 24 h before receiving SA treatments. An empty pcDNA3 plasmid was used as the control.

### Cell migration and invasion assays

Cell migration and invasion assays were performed as described in our previous work [19] with minor modifications. Briefly, for cell migration assay, 2.5 × 10^4^ A549 cells were resuspended in 200 μl of FBS-free RPMI medium containing the appropriate concentration of treatment reagents and plated into the upper chamber of 24-well cell culture inserts with an 8.0-μm pore size transparent polyethylene terephthalate (PET) membrane (Corning Inc., Durham, NC, USA). Meanwhile, 750 μl of RPMI medium containing 10% FBS was placed in the bottom well (Corning Inc., Durham, NC, USA). Cells were allowed to migrate for 24 h in a 37 °C and 5% CO_2_ humidified incubator. After incubation, the inserts were washed twice with PBS, and cells attached to both the upper and lower surfaces of the inserts were fixed with 4% paraformaldehyde (PFA) (Electron Microscopy Sciences, Hatfield, PA, USA) for 10 min and permeabilized with 100% methanol for 20 min before being stained with 0.05% crystal violet (Sigma-Aldrich) for 20 min at room temperature. Subsequently, the upper surface of the inserts was slightly wiped off using a cotton swab to remove nonmigratory cells and the inserts were washed twice with PBS. After air-drying, migrated cells attached to the lower surface of the inserts were subjected to imaging using a Dino-Eye AM423X Digital Microscope Eyepiece Camera (AnMo Electronics Corporation, New Taipei City, Taiwan) connected to a Nikon TMS-F Inverted Phase Contrast Microscope (Nikon, Tokyo, Japan). The bound crystal violet was then eluted from cells with 300 μl of 33% acetic acid (Mallinckrodt Chemicals, Phillipsburg, NJ, USA). The absorbance of the eluted crystal violet was read at 595 nm using a microplate reader (Bio-Rad, Hercules, CA, USA) and determined as a measure of migrated cells. For cell invasion assay, 5 × 10^4^ cells in 200 μl of FBS-free RPMI medium containing the appropriate concentration of treatment reagents were loaded into the upper chamber of 24-well cell culture inserts (Corning Inc., Durham, NC, USA) coated with Matrigel basement membrane matrix (Corning Inc., Bedford, MA, USA), followed by the same procedures as described above.

### Chromatin immunoprecipitation and quantitative real-time PCR

ChIP was performed using the EZ-Magna ChIP™ A/G Chromatin Immunoprecipitation Kit (Merck KGaA, Darmstadt, Germany), following the manufacturer’s instructions. Briefly, 1 × 10^7^ cells were crosslinked in 1% formaldehyde for 10 min and subsequently quenched with 125 mM glycine for 5 min at room temperature. Cells were then placed on ice and washed twice prior to being harvested using a cell scraper. The nuclear fractions separated from cytoplasmic fractions were lysed with the nuclear lysis buffer followed by sonication using a Bioruptor Plus sonicator (Diagenode, Liege, Belgium) at high power for 10 cycles of 30 s ON/OFF at 4 °C. The sheared cross-linked chromatins were then immunoprecipitated with either the rabbit anti-p53 antibody (GTX102965, Genetex) or the rabbit IgG isotype control antibody (GTX35035, Genetex) at 4 °C overnight on a shaker. The positive (immunoprecipitated with anti-RNA polymerase II) and negative (immunoprecipitated with normal mouse IgG) controls were also performed parallelly. The protein-DNA complexes were then washed and eluted from protein A/G magnetic beads. The crosslinks of protein-DNA complexes were reversed, and DNAs purified with spin columns were subjected to qRT-PCR analysis. Genomic DNA sequences in PANX1 and CDKN1A loci retrieved from the University of California Santa Cruz (UCSC) Table Browser (https://genome.ucsc.edu/cgi-bin/hgTables, assembly: Dec. 2013 (GRCh38/hg38)) [78] were used to predict the putative p53 binding sites using the JASPAR program (https://jaspar.genereg.net/) [46]. Specific pairs of primers (Table S5) were designed to amplify target DNA sequence regions located within 2500 base pairs upstream of the transcription start site (TSS) of the PANX1 or CDKN1A (positive control) gene or to amplify the 3′-untranslated region (3′-UTR) of PANX1 (negative control). qRT-PCR was carried out on a StepOnePlus Real-Time PCR system (Applied Biosystems, Foster City, CA, USA) to detect the relative enrichment of p53 on the indicated regions using SYBR Green PCR Master Mix (Applied Biosystems, Foster City, CA, USA). Fold changes of p53 enrichment were expressed as 2^−ΔCt^.

### Caspase-2 activity assay

Caspase-2 activity assays were carried out in black-walled clear-bottom 96-well plates (Corning) using the Caspase-2 Fluorometric Assay Kit (Abcam, Cambridge, UK) according to the manufacturer’s instructions. Briefly, cells were lysed with the cell lysis buffer, and protein concentrations were determined by the Bio-Rad protein assay (Bio-Rad, Hercules, CA, USA). Subsequently, 50 μg of total proteins from each lysed cell sample was mixed with 50 μl of reaction buffer containing 10 mM DTT and 100 µM VDVAD-AFC substrate and the mixture was made up to a total volume of 100 μl per well. The plate was incubated at 37 °C for 1 h and fluorescence was measured at 405 nm and 495 nm wavelengths of excitation and emission, respectively, on a VICTOR Nivo Multimode Plate Reader (PerkinElmer Inc., Shelton, CT, USA).

### Extracellular adenosine triphosphate measurement

The extracellular ATP levels were measured using the luminescent ATP Kit SL (BioThema, Handen, Sweden) according to the manufacturer’s instructions with minor modifications. Briefly, A549 cells were seeded at a density of 8 × 10^3^ cells per well in a nunclon delta-treated 96-well white polystyrene microplate (Thermo Fisher Scientific, Roskilde, Denmark). After overnight culture to allow cell attachment, the original medium was replaced with 60 µl of complete medium containing the desired concentration of treatment reagents, and the cells were incubated for an additional 24 h. At the end of the treatment period, 60 µl of Tris-EDTA buffer (pH 7.75) was gently added to each well followed by adding 20 µl of the luciferase/D-luciferin mixture. Luminescence emission from luciferase-catalyzed luciferin oxidation in the presence of extracellular ATP, corresponding to sample ATP (L_samp_), was measured by a VICTOR3 Multilabel Plate Reader (PerkinElmer Inc., Shelton, CT, USA). To convert the luminescence signal into ATP concentration, 5 µl of a 2 µM ATP Standard was subsequently added to each well to yield a final concentration of approximately 71.4 nM ATP Standard. The luminescence emission after the addition of ATP Standard, corresponding to sample plus Standard ATP (L_samp+std_) was measured, and the concentration of extracellular ATP in each sample (ATP_samp_) was calculated following equation: ATP_samp_ = 71.4 × L_samp_/(L_samp+std_ − L_samp_).

### Flow cytometry

Fluorescence-activated cell sorting (FACS) analyses were used to analyze the cell surface expressions of IL-6Rα on A549 cells, CD14 on THP-1 and THP-1-derived M0 macrophages, or CD80 and CD206 on A549 cells-derived CM-stimulated macrophages using the PE-labeled anti- IL-6Rα (352803; BioLegend, San Diego, CA, USA), the PE-labeled CD14 (12-0149-42; eBioscience, San Diego, CA, USA), or the FITC-labeled CD80 (305205; BioLegend) and APC-labeled CD206 (321109; BioLegend) antibodies, respectively, following the manufacturer’s instructions. Briefly, 100 µl of single-cell suspension (5 × 10^5^ cells) in ice-cold FACS buffer (PBS containing 10% FBS and 0.1% sodium azide) were mixed with 10 µg/ml anti-IL-6Rα-PE, 5 µg/ml anti-CD14-PE, or 20 µg/ml anti-CD80-FITC and 10 µg/ml anti-CD206-APC antibodies. Cells were incubated on ice for 30 min before being washed twice and resuspended in the ice-cold FACS buffer. Flow cytometry was performed using a BD Accuri C6 flow cytometer (BD Biosciences, San Jose, CA, USA), and the data were analyzed by the FlowJo 7.6.1 software (FlowJo LLC, Ashland, OR, USA).

### Differential gene expression analysis

The IL-6 and Panx1 mRNA expression levels in control versus Dox-treated primary human liposarcoma cell cultures were analyzed using the GSE12972 dataset. The IL-6 and Panx1 mRNA expression levels in well, moderately, and poorly differentiated lung adenocarcinoma tumors were analyzed using the GSE68465 dataset. Both datasets were downloaded from Gene Expression Omnibus (GEO) database (https://www.ncbi.nlm.nih.gov/geo/) and the data were log_2_ transformed. Differential gene expression analysis was performed using GraphPad Prism 9 software (GraphPad Inc., San Diego, CA, USA).

### Survival analysis

Correlations between IL-6 or Panx1 gene expression and disease-free survival (DFS) or overall survival (OS) of cancer patients were shown by Kaplan-Meier survival curves plotted using GraphPad Prism 9 software (GraphPad Inc., San Diego, CA, USA). For the correlations of IL-6 or Panx1 gene expression and disease-free survival (DFS) of lung cancer patients, the RNA sequencing (RNA-seq) and patients’ DFS data from the GSE30219 dataset were downloaded from the GEO database (https://www.ncbi.nlm.nih.gov/geo/). For the correlations of IL-6 or Panx1 gene expression and overall survival (OS) of cancer patients, the RNA-Seq by Expectation-Maximization (RSEM) and patients’ overall survival data derived from The Cancer Genome Atlas (TCGA) lung cancer (LUNG) or Pan-Cancer datasets were downloaded from the cBioPortal for Cancer Genomics (https://www.cbioportal.org/) [79, 80]. Patients were divided into high and low IL-6 or Panx1 expression groups based on the median values of IL-6 or Panx1 gene expression, respectively. The statistical significance of the difference between the two groups was determined by log-rank (Mantel-Cox) test.

### Correlation analysis

Gene co-expression correlations between IL-6 and Panx1 or between IL-6 or Panx1 versus genes relevant to DNA damage repair, apoptosis, and epithelial-mesenchymal transition (EMT) were analyzed using the Pearson correlation coefficient (*r*). The correlations of IL-6 and Panx1 gene expression in primary human liposarcomas and that in lung adenocarcinomas were analyzed using the GSE12972 and the GSE68465 datasets, respectively, downloaded from the GEO database (https://www.ncbi.nlm.nih.gov/geo/). For the correlations between IL-6 and Panx1 gene expression or between the gene expression of IL-6 or Panx1 versus genes relevant to DNA damage repair (ATM, ATR, BRCA1, BRCA2, Chk1, and PRKDC), anti-apoptosis (Mcl-1), pro-apoptosis (Bad), EMT stimulation (Snail, Slug, Vimentin, N-cadherin, TWIST1, TWIST2, and MMP9), and EMT inhibition (E-Cadherin, EPCAM, TJP3, and Occludin) in lung cancer patients, we used the RSEM data from the TCGA LUNG cohort downloaded from the cBioPortal for Cancer Genomics (https://www.cbioportal.org/) [79, 80]. All RNA-seq data were log_2_ transformed. The heatmaps of correlation coefficients (*r*) were generated by GraphPad Prism 9 software (GraphPad Inc., San Diego, CA, USA).

### Preparation of conditioned medium

To obtain the conditioned medium (CM) of SA- or Dox-treated A549 cells, cells were seeded at a density of 6 × 10^5^ cells per 10-cm cell culture dish (Corning Inc., Durham, NC, USA) in complete RPMI medium at 37 °C and 5% CO_2_ in a humidified incubator. After 24 h of attachment, cells were treated with SA or Dox diluted in complete RPMI medium at desired concentrations for an additional 24 h. Subsequently, the SA- or Dox-treated cells were washed twice with fresh medium to eliminate residual SA or Dox and the medium was then replaced with serum-free RPMI medium. IL-6 gene expression was measured at various time points during the incubation (Fig. S6A). After 24 h of incubation, the CM from control or SA/Dox-treated cells was harvested and centrifuged at 1000 × *g* for 5 min at 4 °C to remove cell debris. The supernatant was collected. IL-6 concentration in the CM was measured (Fig. S6B) and the CM was stored at −80 °C until use.

### Macrophage differentiation and polarization

Human monocytic THP-1 cells were seeded at a density of 2.5 × 10^6^ cells per 6-cm culture dish (Corning Inc., Durham, NC, USA) in a complete RPMI medium containing 10 ng/ml phorbol 12-myristate 13-acetate (PMA) (Sigma-Aldrich) at 37 °C and 5% CO_2_ to allow differentiation of monocytes into macrophage-like cells (Fig. S5A). After 24 h of incubation, primed macrophages were washed twice with fresh medium to remove residual PMA, and cells were cultured in serum-free RPMI medium for an additional 24 h. Cells at this stage were considered differentiated nonpolarized (M0) macrophages. The positive control M1-polarized macrophages were obtained by co-treating M0 macrophages with 1 μg/ml lipopolysaccharide (LPS) (Sigma-Aldrich) and 20 ng/ml recombinant human IFN-γ (PeproTech, Cranbury, NJ, USA) for 24 h. Meanwhile, the positive control M2-polarized macrophages were generated by treating M0 macrophages with 20 ng/ml recombinant human IL-4 (PeproTech) for 24 h. To investigate the effects of SA- or Dox-treated A549 cells on macrophage polarization, M0 macrophages were stimulated with the aforementioned conditioned medium from SA- or Dox-treated cells for 24 h. Subsequently, the polarized macrophages were harvested and subjected to qRT-PCR analysis of the gene expressions (Fig. 6A–F) or flow cytometric analysis of the cell surface expressions (Fig. 6G) of the M1- and M2-associated macrophage markers. Alternatively, polarized macrophages were washed twice with fresh medium and cultured in complete RPMI medium for another 24 h (Fig. S5A). The conditioned medium from polarized macrophages was then harvested and centrifuged at 1000 × *g* for 5 min at 4 °C to remove cell debris. The supernatant was finally collected and stored at −80 °C until being used to treat cancer cells (Fig. 6H–K).

### Co-culture of A549 cells and THP-1-derived macrophages

To study the role of IL-6 secreted by cancer cells in macrophage polarization, control or IL-6-silenced A549 cells (1 × 10^5^ cells/well) were resuspended in 500 μl of serum-free RPMI medium containing appropriate SA or Dox concentrations and seeded into the upper chamber of 6-well cell culture inserts with an 8.0-μm pore size transparent PET membrane (Corning Inc., Durham, NC, USA) (Fig. 7A). The inserts containing cancer cells were then placed into wells of a 6-well cell culture plate (Corning Inc., Wujiang, Jiangsu, China) containing THP-1-derived M0 macrophages (1 × 10^6^ cells/well in 1.5 ml of serum-free RPMI medium), which had been previously prepared as described above. A similar experiment was conducted with 1.5 ml of serum-free RPMI medium alone instead of THP-1-derived M0 macrophages plated in the bottom wells and the culture medium collected from the bottom wells after 24 h of incubation was subjected to enzyme-linked immunosorbent assay (ELISA) measurements of the IL-6 protein concentration secreted by cancer cells cultured in the upper chambers (Fig. S6C). Co-cultures of A549 cells and M0 macrophages were maintained for 24 h to induce macrophage polarization before the polarized macrophages were harvested and subjected to qRT-PCR analysis of the gene expressions (Fig. S5H–J) or flow cytometric analysis of the cell surface expressions (Fig. 7B) of the M1- and M2-associated markers. A control experiment was conducted with RPMI medium alone instead of A549 cells added to the upper chamber (Fig. 7A).

To evaluate the effects of the polarized macrophages stimulated by SA/Dox-treated A549 cells-secreted IL-6 on the migratory capacity of cancer cells (Fig. 7C, D), control or IL-6-silenced A549 cells (2.5 × 10^4^ cells/well in 200 μl of FBS-free RPMI medium) treated with appropriate concentrations of SA or Dox were plated into the upper chamber of 24-well cell culture inserts with an 8.0-μm pore size transparent PET membrane (Corning Inc., Durham, NC, USA) and co-cultured with M0 macrophages (2.5 × 10^5^ cells/well in 750 μl of FBS-free RPMI medium) pre-prepared in the bottom well of a 24-well cell culture plate (Corning Inc., Durham, NC, USA) as described above. After 24 h of co-culture, the inserts containing control or IL-6-silenced A549 cells were removed, the polarized macrophages in the bottom well were washed twice with fresh medium, and the medium in the bottom well was replaced with 750 μl of RPMI medium containing 10% FBS. A control experiment was conducted with RPMI medium alone instead of control or IL-6-silenced A549 cells added to the upper chamber. Finally, parental A549 cells (2.5 × 10^4^ cells/well) were resuspended in 200 μl of FBS-free RPMI medium and loaded into the upper chamber of new 24-well cell culture inserts (Corning Inc., Durham, NC, USA). These inserts were subsequently placed into wells of the 24-well cell culture plate (Corning Inc., Durham, NC, USA) containing polarized macrophages pre-stimulated with control or IL-6-silenced A549 cells with and without SA/Dox treatment as described above. The migratory ability of parental A549 cells in the upper chamber was analyzed 24 h post-incubation (Fig. 7C). To measure cell invasiveness (Fig. 7D), parental A549 cells (5 × 10^4^ cells/well) in 200 μl of FBS-free RPMI medium were plated into the upper chamber of 24-well cell culture inserts (Corning Inc., Durham, NC, USA) coated with Matrigel basement membrane matrix (Corning Inc., Bedford, MA, USA), followed by the same procedures as described above.

### Software and statistical analysis

GraphPad Prism 9 software (GraphPad Inc., San Diego, CA, USA) was used to perform all statistical analyses and create graphs. All data are shown as means ± standard deviation (SD) of at least three independent experiments. Samples were randomly allocated to different treatment conditions. Representative areas of cell culture chambers were randomly selected for imaging with the microscope. Detailed information regarding the statistical test and sample size (*n*) applied for each experiment were stated in the figure legends. The *p-*value, which is ≤0.05, was considered statistically significant. Statistical significance was denoted by asterisks (*p ≤ 0.05, **p ≤ 0.01, and ***p ≤ 0.001). ns denoted no significance (*p* > 0.05). All composite figures were assembled in Adobe Illustrator.

### Supplementary information

Supplementary Information

Supplementary Figure S1

Supplementary Figure S2

Supplementary Figure S3

Supplementary Figure S4

Supplementary Figure S5

Supplementary Figure S6

Full and Uncropped Western Blots

## Data Availability

All data generated or analyzed during this study are available from the corresponding authors upon reasonable request.
