# Supplementary material for: Tumor suppressor p53 mediates interleukin-6 expression to enable cancer cell evasion of genotoxic stress
Source: Cell Death Discov. 2023 Sep 11;9:340. doi: 10.1038/s41420-023-01638-0 (PMC10495329; doi:10.1038/s41420-023-01638-0)
Supplement: Supplementary file 1 — Supplementary Information [file 41420_2023_1638_MOESM1_ESM.pdf]

Supplementary Information

Tumor Suppressor p53 Mediates Interleukin-6 Expression to Enable Cancer Cell Evasion of Genotoxic Stress

Trinh T. T. Phan[1,†], Nam V. Truong[2,†], Wen-Guey Wu[2], Yi-Chun Su[1], Tzu-Sheng Hsu[1,*], and Lih-Yuan Lin[1,*]

[1] Institute of Molecular and Cellular Biology, College of Life Sciences and Medicine, National Tsing Hua University, Hsinchu 300044, Taiwan R.O.C

[2] Institute of Bioinformatics and Structural Biology, College of Life Sciences and Medicine, National Tsing Hua University, Hsinchu 300044, Taiwan R.O.C

[†] These authors contributed equally to this work.

***Corresponding authors:** Lih-Yuan Lin, Tzu-Sheng Hsu

Email: lylin@life.nthu.edu.tw (LYL), tshsu@life.nthu.edu.tw (TSH)

This PDF file includes:

Supplementary Figure Legends

Supplementary Tables S1 to S5

Supplementary Figure Legends

Fig. S1 IL-6 is induced by genotoxic stress and drives cancer cell tolerance to genotoxic treatments. **A-C** IL-6 gene expression in A549 or H1299 cells treated with increasing concentrations of (A) sodium arsenite (SA), (B) doxorubicin (Dox), or (C) cisplatin (CisPt) for 24 h was measured by quantitative real-time PCR (qRT-PCR). **D** IL-6 mRNA levels in primary human liposarcoma cell cultures were analyzed using the GSE12972 dataset and their log_2-transformed values were compared between control (n = 19) and paired Dox-treated (n = 19) cells. **E** The concentration of IL-6 present in the culture supernatants of A549 or H1299 cells treated with the indicated concentrations of CisPt for 24 h was determined by ELISA. **F**, **G** Flow cytometric analysis of the cell surface expression of IL-6Rα in A549 cells treated with increasing concentrations of (F) SA or (G) CisPt for 24 h. Cells were stained with PE-conjugated antibody against IL-6Rα. **H** IL-6 gene expression in control (scramble) and IL-6-silenced (shIL-6-1 and shIL-6-2) A549 cells untreated or treated with 20 μM SA for 24 h was measured by qRT-PCR. **I** IL-6 protein concentration in the culture supernatants of scramble, shIL-6-1, and shIL-6-2 A549 cells untreated or treated with 20 μM SA for 24 h was determined by ELISA. **J** IL-6 gene expression in control (siCtrl) and IL-6-silenced (siIL-6) A549 cells untreated or treated with 20 μM SA, 0.5 μM Dox, or 20 μM CisPt for 24 h was measured by qRT-PCR. **K** IL-6 protein concentration in the culture supernatants of siCtrl and siIL-6 A549 cells untreated or treated with 20 μM SA, 0.5 μM Dox, or 20 μM CisPt for 24 h was determined by ELISA. **L** Dose-response curves showing the survival of siCtrl and siIL-6 A549 cells in response to increasing concentrations of CisPt treatment for 24 h. **M** The IC_{50} values of CisPt against siCtrl and siIL-6 A549 cells were calculated from the nonlinear regression curves in Fig. S1L. Cell viability was measured by MTT assays. Error bars represent mean ± SD, n = 3. Statistical analysis was performed using unpaired two-tailed Student's *t*-test (A-C, E-G, M), paired two-tailed Student's *t*-test (D), or two-way ANOVA with Tukey's multiple comparisons test (H-K). *$p \leq 0.05$; **$p \leq 0.01$; ***$p \leq 0.001$; ns, not significant.

Fig. S2 IL-6 silencing heightens genotoxic stress-induced cell death in human breast cancer MCF-7 and cervical cancer HeLa cells. **A**, **H** IL-6 gene expression in control (siCtrl) and IL-6-silenced (siIL-6) (A) MCF-7 or (H) Hela cells untreated or treated with 20 μM SA, 0.5 μM Dox, or 20 μM CisPt for 24 h was measured by qRT-PCR. **B**, **D**, **F**, **I**, **K**, **M** Dose-response curves showing the survival of siCtrl and siIL-6 (B, D, F) MCF-7 or (I, K, M) Hela cells in response to increasing concentrations of (B, I) SA, (D, K) Dox, or (F, M) CisPt treatment for 24 h. **C**, **E**, **G**, **J**, **L**, **N** The IC_{50} values of (C, J) SA, (E, L) Dox, or (G, N) CisPt against siCtrl and siIL-6 (C, E, G) MCF-7 or (J, L, N) Hela cells were calculated from the nonlinear regression curves in Fig. S2B, D, F, I, K, M, respectively. Cell viability was measured by MTT assays. Error bars represent mean ± SD, n = 3. Statistical analysis was performed using two-way ANOVA with Tukey's multiple comparisons test (A, H) or unpaired two-tailed Student's *t*-test (C, E, G, J, L, N). $*p \leq 0.05$; $**p \leq 0.01$; $***p \leq 0.001$.

Fig. S3 IL-6 mitigates genotoxic stress-induced cell death. **A**, **C**, **E** Dose-response curves showing the survival of control and 50 or 20 ng/ml IL-6-pretreated (A) A549 or (C, E) H1299 cells, respectively, in response to increasing concentrations of (A, C) SA or (E) CisPt treatment for 24 h. **B**, **D**, **F** The IC_{50} values of (B, D) SA or (F) CisPt against control and 50 or 20 ng/ml IL-6-pretreated (B) A549 or (D, F) H1299 cells were calculated from the nonlinear regression curves in Fig. S3A, C, E, respectively. **G**, **I** Dose-response curves showing the survival of siCtrl, siIL-6, and siIL-6+IL-6 A549 cells in response to increasing concentrations of (G) SA or (I) Dox treatment for 24 h. **H**, **J** The IC_{50} values of (H) SA or (J) Dox against siCtrl, siIL-6, and siIL-6+IL-6 A549 cells were calculated from the nonlinear regression curves in Fig. S3G and S3I, respectively. Cell viability was measured by MTT assays. **K**-**M** The mRNA levels of the anti-apoptotic genes (K)

Bcl-xL, (L) Mcl-1, and (M) Bcl-2 in A549 cells treated with increasing concentrations of SA for 24 h were measured by qRT-PCR. **N** The proliferation of scramble, shIL-6-1, and shIL-6-2 A549 cells untreated or treated with 20 μM SA for 24 h was assessed by manual cell counting with a hemocytometer. Error bars represent mean ± SD, n = 3. Statistical analysis was performed using unpaired two-tailed Student's *t*-test (B, D, F, K-M) or one-way ANOVA with Tukey's multiple comparisons test (H, J). *$p \le 0.05$; **$p \le 0.01$; ***$p \le 0.001$; ns, not significant.

Fig. S4 p53 silencing attenuates IL-6 expression. qRT-PCR analysis of (A, C) p53 and (B, D) IL-6 gene expressions in control (siCtrl) and p53-silenced (sip53) (A, B) MCF-7 or (C, D) Hela cells untreated or treated with 0.5 μM Dox for 24 h. Error bars represent mean ± SD, n = 3. Statistical analysis was performed using two-way ANOVA with Tukey's multiple comparisons test. *$p \le 0.05$; **$p \le 0.01$; ***$p \le 0.001$; ns, not significant.

Fig. S5 Genotoxic-treated cancer cells secrete IL-6 to enable alternative (M2) macrophage polarization. **A** THP-1 monocytes were differentiated into M0 macrophages by treatment with 10 ng/ml PMA for 24 h followed by culture in serum-free RPMI medium for an additional 24 h and M0 macrophages were then stimulated with conditioned media from untreated or SA/Dox-treated A549 cells to induce macrophage polarization as depicted. **B** Phase-contrast imaging of THP-1 monocytes and 10 ng/ml PMA-primed macrophages before and after being cultured in serum-free RPMI medium for 24 h. Scale bar: 100 μm. **C-F** The mRNA levels of the macrophage markers (C) CD14, (D) CD68, (E) IL-1β, and (F) TNF-α were analyzed in THP-1 monocytes and THP-1-derived M0 macrophages using qRT-PCR. **G** Flow cytometric analysis of the cell surface expression of the macrophage marker CD14 in THP-1 monocytes and THP-1-derived M0 macrophages. Cells were stained with PE-conjugated antibody against CD14. **H-J** The mRNA

levels of (H, I) M2- (CD206 and CD163) or (J) M1-associated (CD80) macrophage markers were analyzed in THP-1-derived macrophages co-cultured with RPMI, control (scramble), or IL-6-silenced (shIL-6-1 and shIL-6-2) A549 cells with and without 20 μM SA or 0.1 μM Dox treatment for 24 h using qRT-PCR. Error bars represent mean ± SD, n = 3. Statistical analysis was performed using unpaired two-tailed Student's *t*-test (C-G) or two-way ANOVA with Tukey's multiple comparisons test (H-J). *$p \leq 0.05$; **$p \leq 0.01$; ***$p \leq 0.001$; ns, not significant.

Fig. S6 IL-6 expression in SA-treated A549 cells after SA removal and IL-6 concentration in macrophage-stimulating conditioned media. **A** qRT-PCR analysis of IL-6 mRNA levels in A549 cells after being treated with 20 μM SA for 24 h and cultured in SA-free RPMI medium for the indicated time periods (1, 3, 6, 12, and 24 h). **B** The concentration of IL-6 present in the culture supernatants of A549 cells after being treated with SA (20 or 40 μM) or Dox (0.1 or 0.5 μM) for 24 h and cultured in SA/Dox-free RPMI medium for an additional 24 h. **C** The concentration of IL-6 present in the conditioned medium collected from the bottom wells of a 24-well cell culture plate was determined by ELISA. The upper chamber was plated with control (scramble) or IL-6-silenced (shIL-6-1 and shIL-6-2) A549 cells with and without 20 μM SA or 0.1 μM Dox treatment for 24 h. Error bars represent mean ± SD, n = 3. Statistical analysis was performed using one-way ANOVA with Tukey's multiple comparisons test (A, B) or two-way ANOVA with Tukey's multiple comparisons test (C). *$p \leq 0.05$; **$p \leq 0.01$; ***$p \leq 0.001$; ns, not significant.

Supplementary Tables

Table S1. IC_{50} of SA, Dox, or CisPt under various treatment conditions

Figure	Cell line	Treatment	IC_{50} of SA (μM)	IC_{50} of Dox (μM)	IC_{50} of CisPt (μM)
1H, I	A549	Scramble	256.90 ± 11.75		
		shIL-6-1	183.03 ± 2.72		
		shIL-6-2	192.80 ± 14.64		
1J ,K	A549	Scramble		112.05 ± 25.18	
		shIL-6 -1		11.60 ± 2.71	
		shIL-6 -2		28.09 ± 0.93	
S1L, M	A549	siCtrl			113.09 ± 12.73
		siIL-6			19.27 ± 0.39
S2B, C	MCF-7	siCtrl	47.45 ± 2.12		
		siIL-6	23.45 ± 5.06		
S2D, E	MCF-7	siCtrl		0.81 ± 0.12	
		siIL-6		0.44 ± 0.08	
S2F, G	MCF-7	siCtrl			214.97 ± 11.37
		siIL-6			107.30 ± 3.03
S2I, J	Hela	siCtrl	70.72 ± 8.53		
		siIL-6	33.69 ± 3.46		
S2K, L	Hela	siCtrl		24.80 ± 7.29	
		siIL-6		1.89 ± 0.93	
S2M, N	Hela	siCtrl			62.63 ± 2.60
		siIL-6			32.72 ± 2.82
S3A, B	A549	Vehicle	254.20 ± 11.37		
		IL-6	392.87 ± 84.06		

S3C, D	H1299	Vehicle	17.02 ± 0.76		
		IL-6	22.27 ± 2.85		
S3E, F	H1299	Vehicle			48.67 ± 8.22
		IL-6			76.80 ± 2.73
S3G, H	A549	siCtrl	258.87 ± 12.25		
		siIL-6	158.33 ± 11.40		
		siIL-6+IL-6	195.57 ± 14.62		
S3I, J	A549	siCtrl		93.91 ± 3.66	
		siIL-6		2.41 ± 0.29	
		siIL-6+IL-6		13.07 ± 0.80	
3G, H	A549	Vehicle	249.40 ± 1.51		
		BAPTA-AM	138.00 ± 11.15		
3N, O	A549	Vehicle	264.27 ± 9.24		
		LY294002	124.73 ± 18.16		
5M, N	A549	siCtrl	249.80 ± 21.85		
		sip53	148.30 ± 6.12		
6H, I	A549	RPMI	252.27 ± 11.25		
		Ctrl CM	38.30 ± 0.80		
		SA CM	60.73 ± 9.76		
6J, K	H1299	RPMI	16.82 ± 1.10		
		Ctrl CM	11.83 ± 1.19		
		SA CM	17.06 ± 2.85		

Table S2. List of primers used for qRT-PCR

Target protein	Sequence (5'-3')
IL-6	Forward: AGACAGCCACTCACCTCTTCAG
	Reverse: TTCTGCCAGTGCCTCTTTGCTG
Panx1	Forward: CCACGGAGTACGTGTTCTCG
	Reverse: CCGCCCAGCAATATGAATCC
Bcl-2	Forward: ATGTGTGTGGAGAGCGTCAA
	Reverse: ACAGTTCCACAAAGGCATCC
Bcl-xL	Forward: GAGCTGGTGGTTGACTTTCTC
	Reverse: TCCATCTCCGATTCAGTCCCT
Mcl-1	Forward: TAAGGACAAAACGGGACTGG
	Reverse: ACCAGCTCCTACTCCAGCAA
p53	Forward: AAGGAAATTTGCGTGTGGAGT
	Reverse: AAAGCTGTTCCGTCCCAGTA
CD206	Forward: GGGAAAGGTTACCCTGGTGG
	Reverse: GTCAAGGAAGGGTCGGATCG
CD163	Forward: TTTGTCAACTTGAGTCCCTTCAC
	Reverse: TCCCGCTACACTTGTTTTCAC
CCL18	Forward: TGGCAG ATTCCACAAAAGTTCA
	Reverse: GGATGACACCTGGCTTGGG
CD80	Forward: GGCCCGAGTACAAGAACCG
	Reverse: TCGTATGTGCCCTCGTCAGAT
CXCL10	Forward: TGTACGCTGTACCTGCATCA
	Reverse: GGACAAAATTGGCTTGCAGGA

IL-1β	Forward: CTCGCCAGTGAAATGATGGCT
	Reverse: GTCGGAGATTCGTAGCTGGAT
CD14	Forward: CTGCAACTTCTCCGAACCTC
	Reverse: CCAGTAGCTGAGCAGGAACC
CD68	Forward: CTTCTCTCATTCCCCTATGGACA
	Reverse: GAAGGACACATTGTACTCCACC
TNF-α	Forward: TTCTGCCTGCTGCACTTTGGA
	Reverse: TTGATGGCAGAGAGGAGGTTG
UBC	Forward: CTGGAAGATGGTCGTACCCTG
	Reverse: GGTCTTGCCAGTGAGTGTCT
GAPDH	Forward: TGCACCACCAACTGCTTAGC
	Reverse: GGCATGGACTGTGGTCATGAG

Table S3. shRNA sequences

shRNA name	Clone ID	Oligo sequence (5’-3’)
Scramble	ASN0000000004	CCGGCCTAAGGTTAAGTCGCCCTCGCTCGAGCGA GGGCGACTTAACCTTAGGTTTTT
shIL-6-1	TRCN0000059207	CCGGCAGAACGAATTGACAAACAAACTCGAGTTT GTTTGTCAATTCGTTCTGTTTTTG
shIL-6-2	TRCN0000059206	CCGGGACATGTAACAAGAGTAACATCTCGAGATG TTACTCTTGTTACATGTCTTTTTG

Table S4. siRNA sequences

Target protein	siRNA ID	Target sequence (5'-3')
p53	VHS40366	Sense: CCAUCCACUACAACUACAUGUGUAA
		Antisense: UUACACAUGUAGUUGUAGUGGAUGG
Caspase-2	HSS141454	Sense: UGCACGUGGCCGACAUGCUGGUUAA
		Antisense: UUAACCAGCAUGUCGGCCACGUGCA
IL-6	s7311	Sense: GAACGAAUUGACAAACAAAtt
		Antisense: UUUGUUUGUCAAUUCGUUCtg

Table S5. List of primers used for ChIP-qPCR

Gene locus	Target region	Sequence (5’-3’)
PANX1	ChIP 1 (-1911 to -1768)	Forward: GGGAGAATTTCACAAAGGGACC
		Reverse: CTTCCCACCCAAAACACTCC
	ChIP 2 (-1441 to -1329)	Forward: TGGTTATGGCAGCTGTGAGA
		Reverse: CCTGAACTCCATCTTCCCCA
	ChIP 3 (-1277 to -1169)	Forward: TATGAGCCATCTACGCGGAG
		Reverse: GGGTCCACAGATACAAGCCT
	ChIP 4 (-817 to -652)	Forward: CCTGTGTACCATACCCTCCG
		Reverse: GGAAGCTAAATGTGAGGATCCA
	ChIP 5 (-622 to -488)	Forward: CCTTAATTTCCGTGGACACTGG
		Reverse: GAGCCAGCAGAGAGAGGG
	ChIP 6 (-218 to -119)	Forward: GCCATCCCATCCACAGAAAG
		Reverse: GAAATTCTCCCGTCCCTCCC
	3’-UTR (+2675 to +2791)	Forward: TGCTTCATATGGCGTGCTTG
		Reverse: CCAAATGGCCTCAATTCGGA
CDKN1A	-2292 to -2169	Forward: GGCTCTGATTGGCTTTCTGG
		Reverse: TCCTACCATCCCCTTCCTCA
