# Supplementary figures and images for: Tumor suppressor p53 mediates interleukin-6 expression to enable cancer cell evasion of genotoxic stress
Source: Cell Death Discov. 2023 Sep 11;9:340. doi: 10.1038/s41420-023-01638-0 (PMC10495329; doi:10.1038/s41420-023-01638-0)

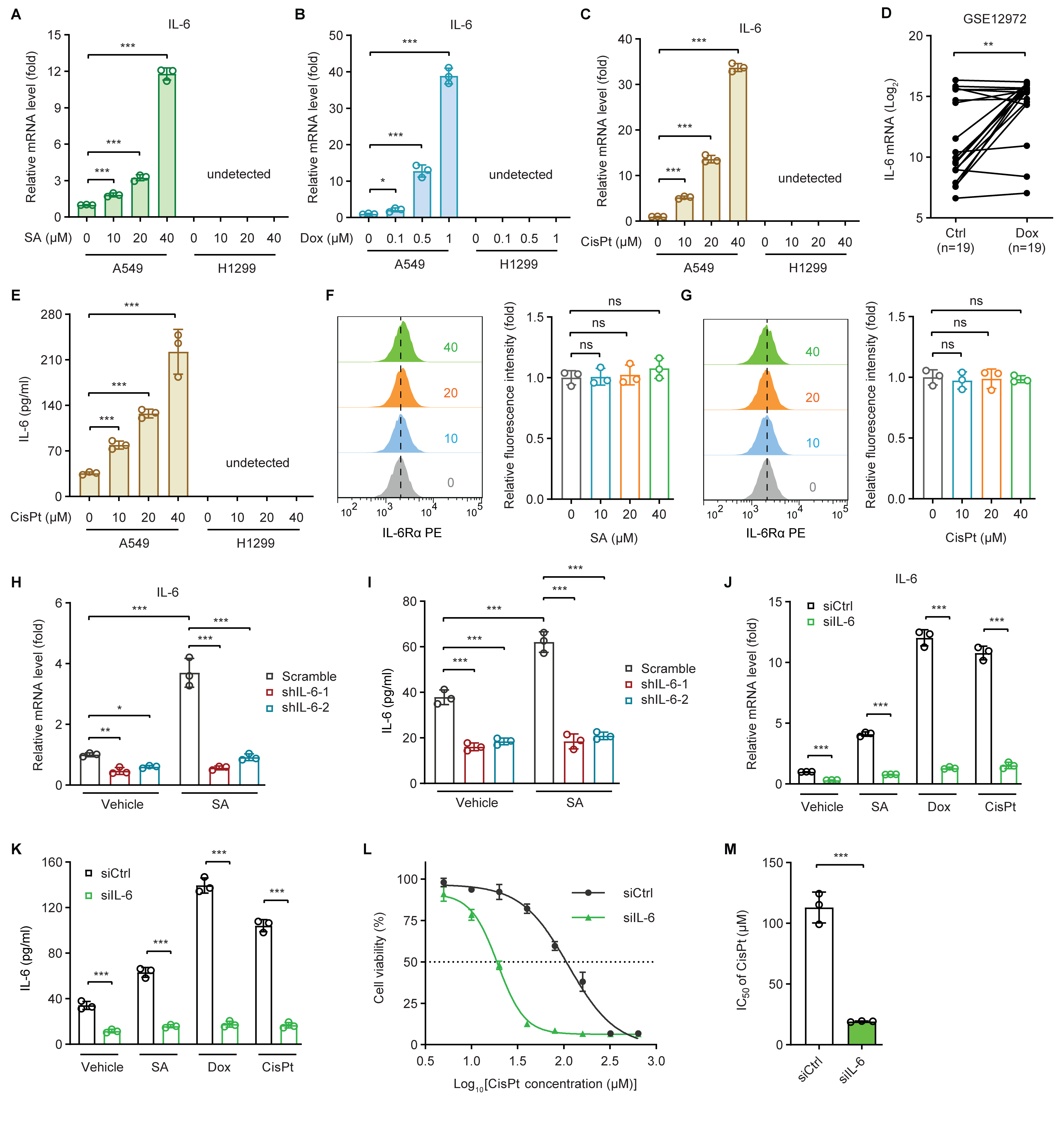

Supplement: Supplementary file 2 — Supplementary Figure S1 [file 41420_2023_1638_MOESM2_ESM.tif]

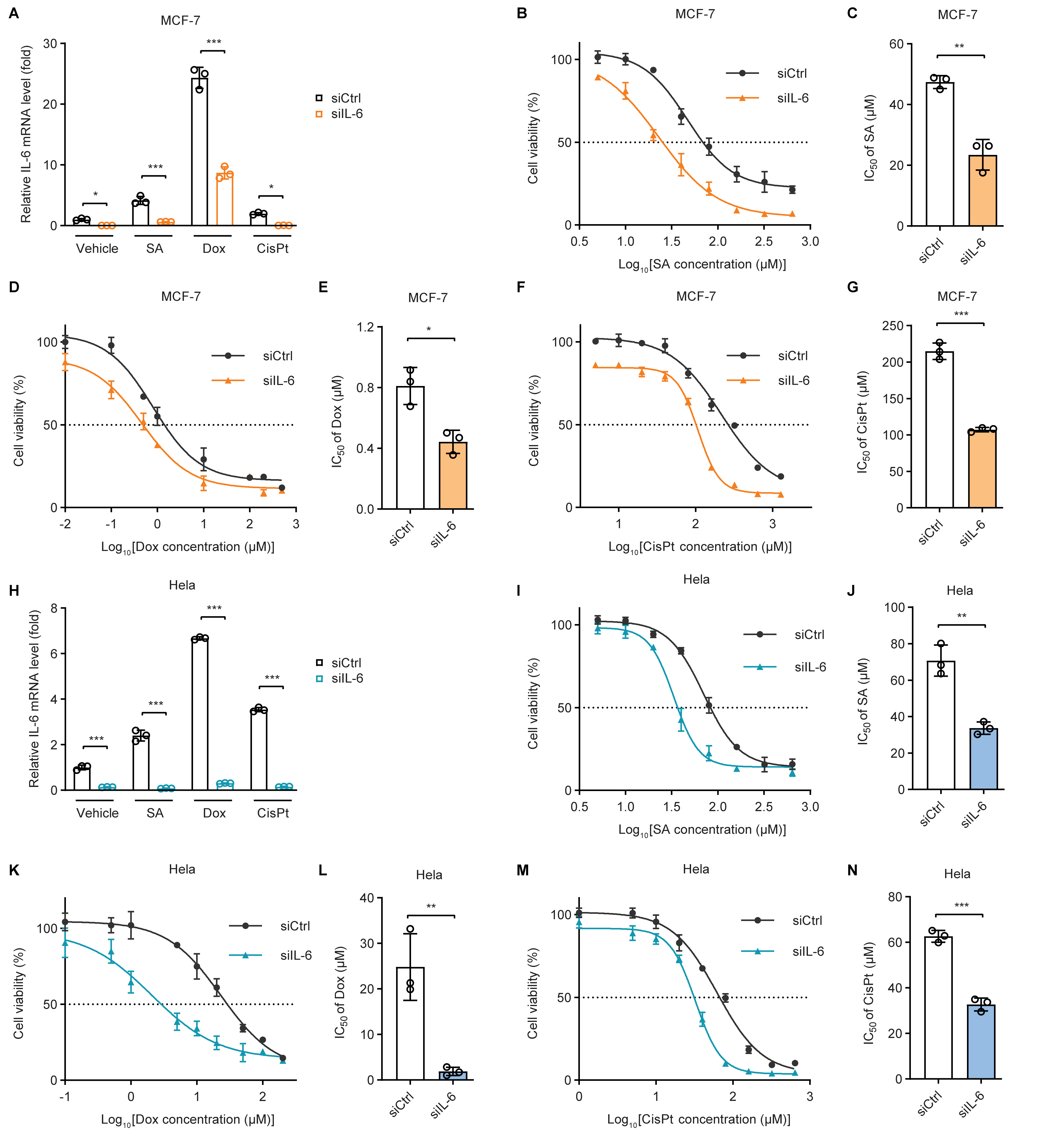

Supplement: Supplementary file 3 — Supplementary Figure S2 [file 41420_2023_1638_MOESM3_ESM.tif]

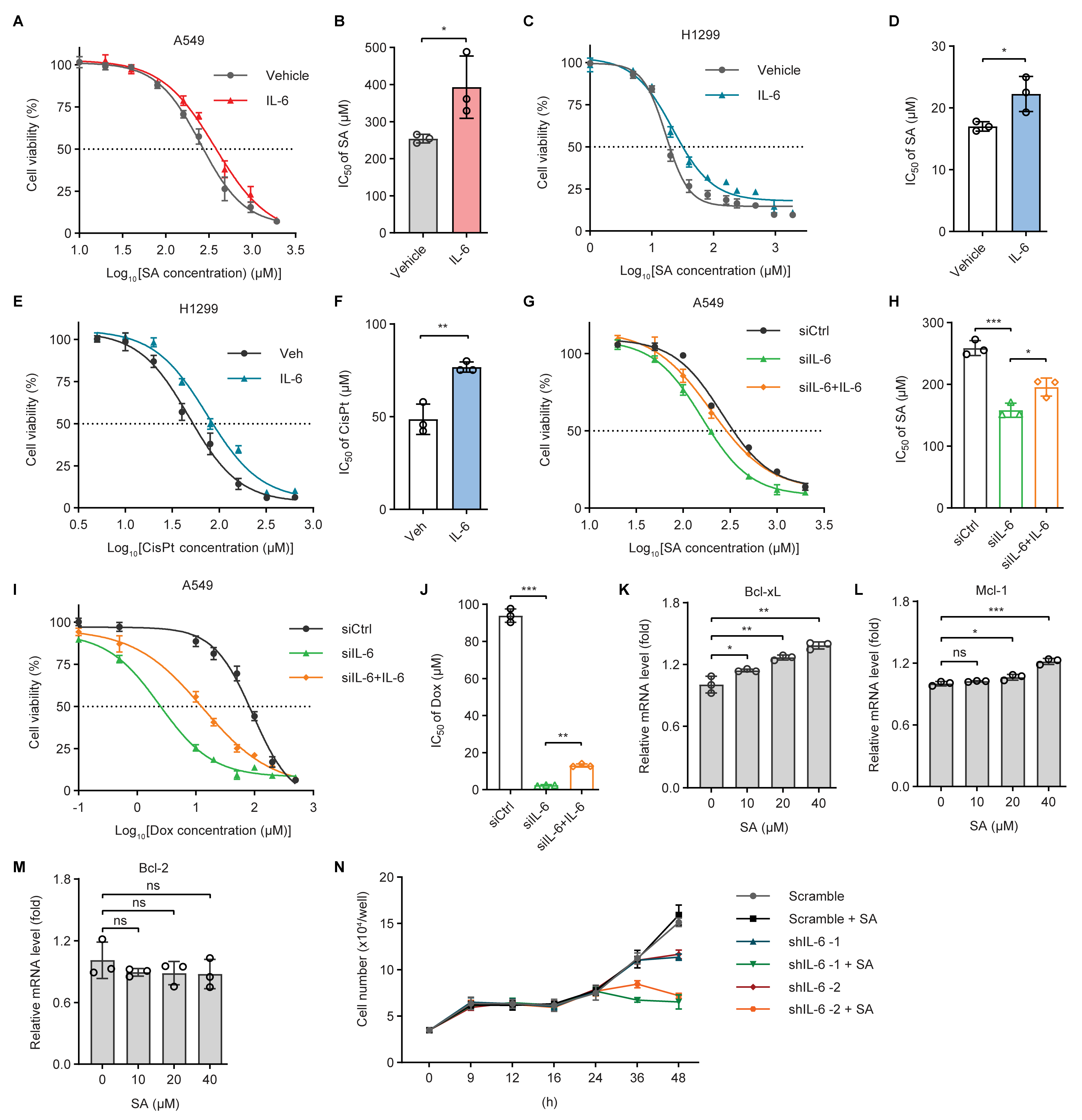

Supplement: Supplementary file 4 — Supplementary Figure S3 [file 41420_2023_1638_MOESM4_ESM.tif]

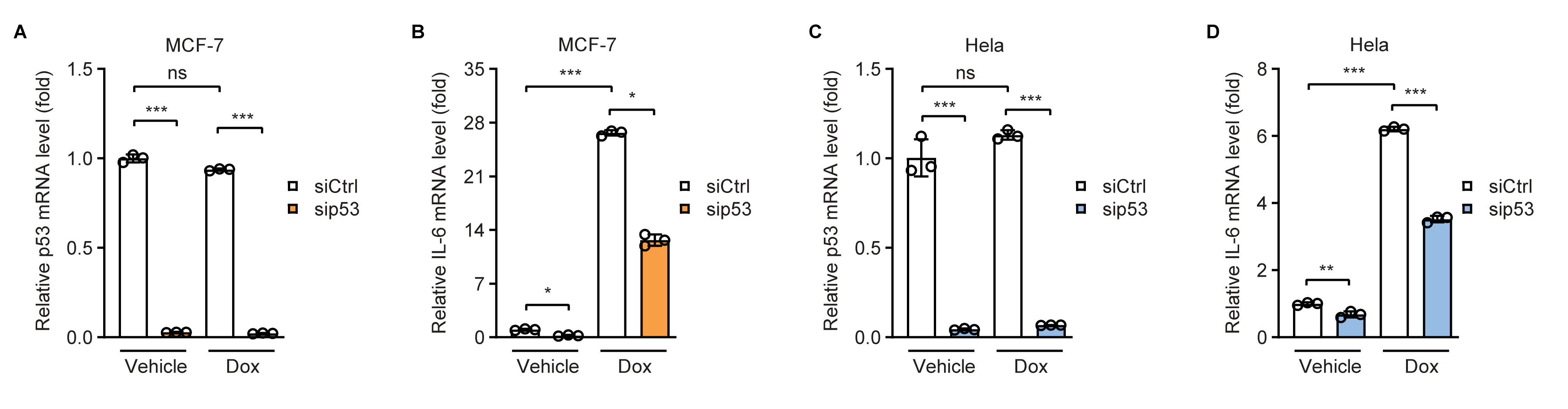

Supplement: Supplementary file 5 — Supplementary Figure S4 [file 41420_2023_1638_MOESM5_ESM.tif]

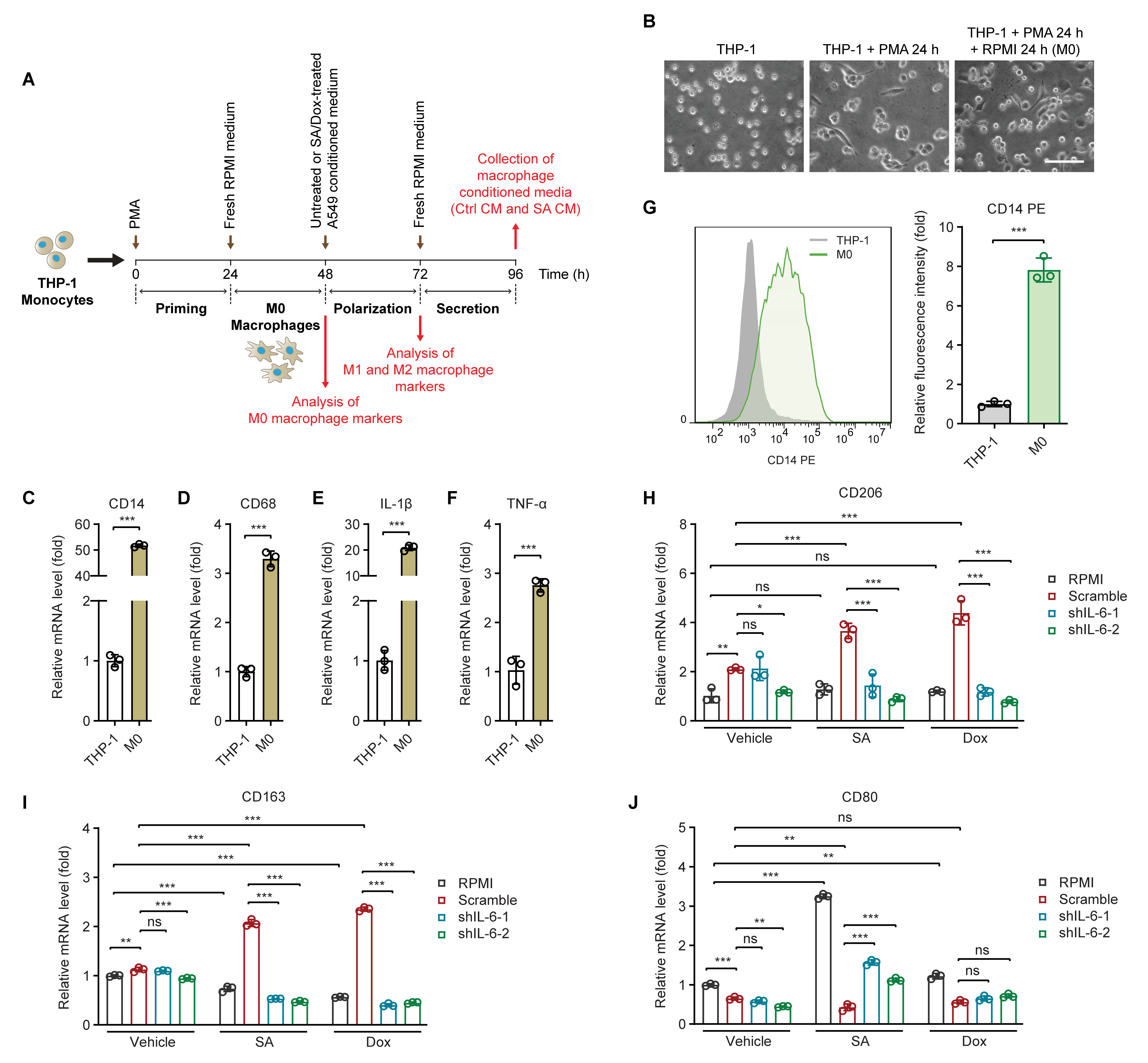

Supplement: Supplementary file 6 — Supplementary Figure S5 [file 41420_2023_1638_MOESM6_ESM.tif]

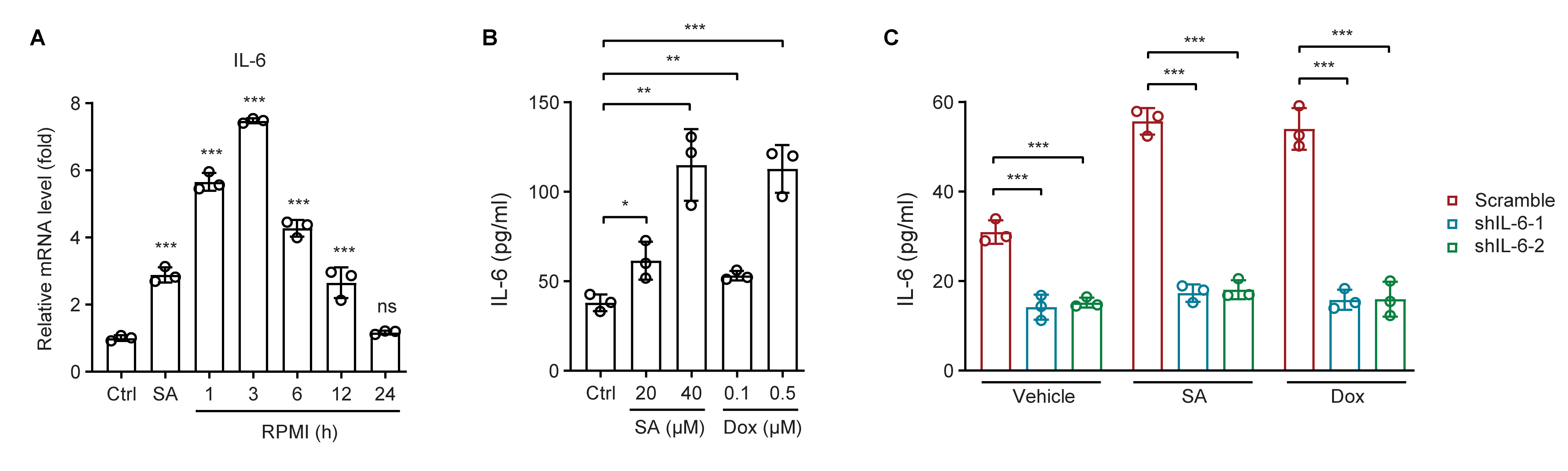

Supplement: Supplementary file 7 — Supplementary Figure S6 [file 41420_2023_1638_MOESM7_ESM.tif]

**Fig. 1G**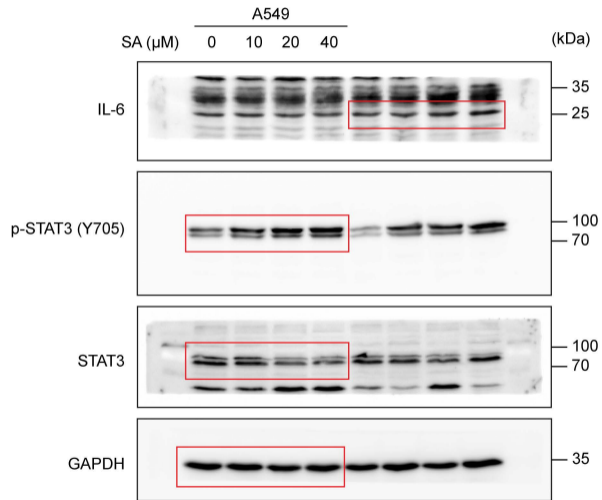**Fig. 1L**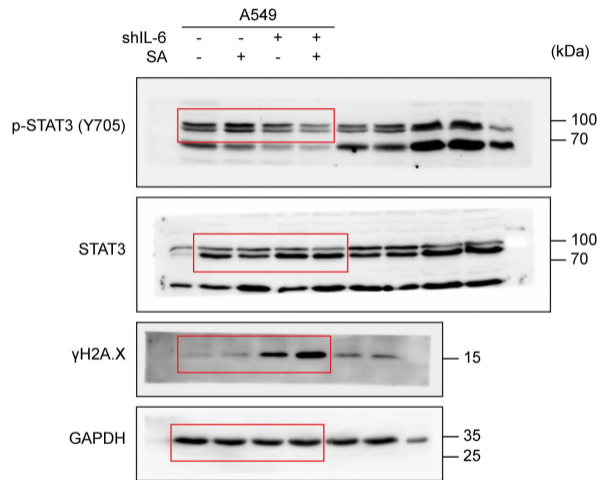

Fig. 3J

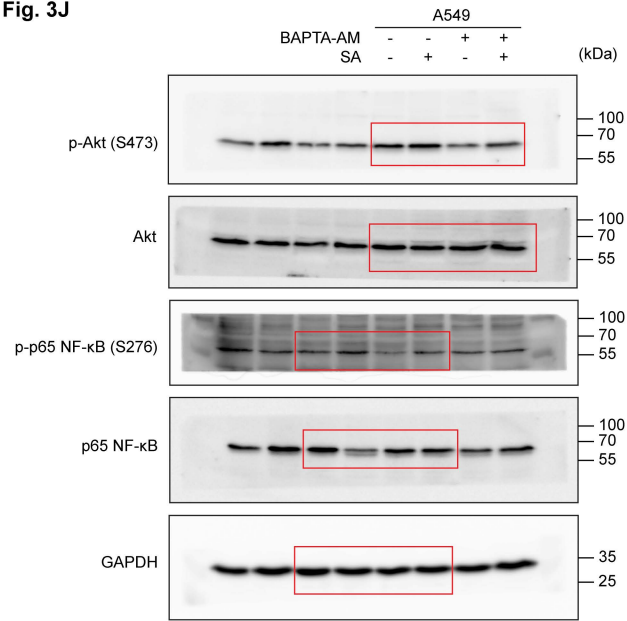

Fig. 3M

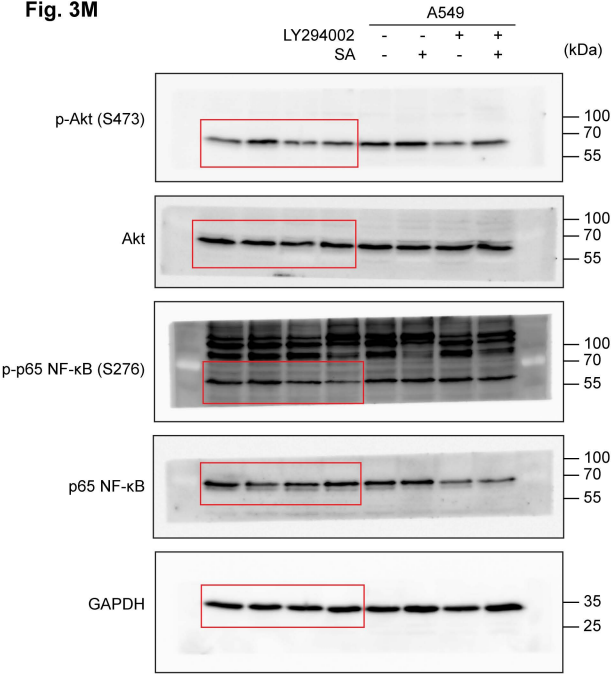

**Fig. 4C**

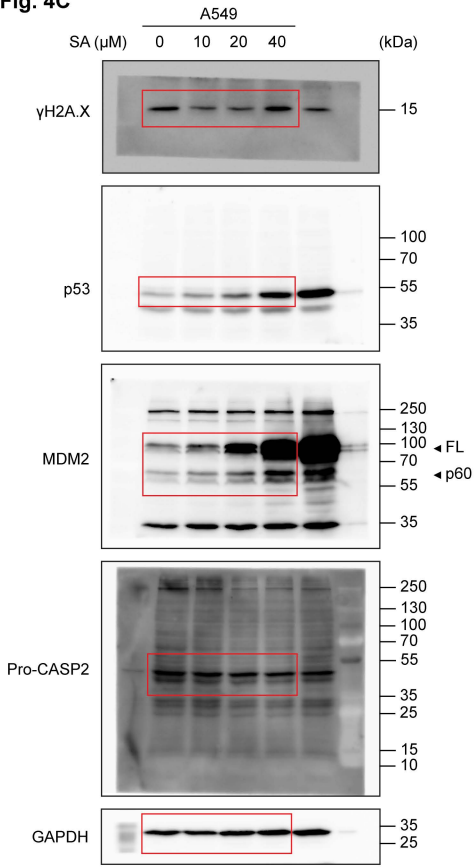

**Fig. 4G**

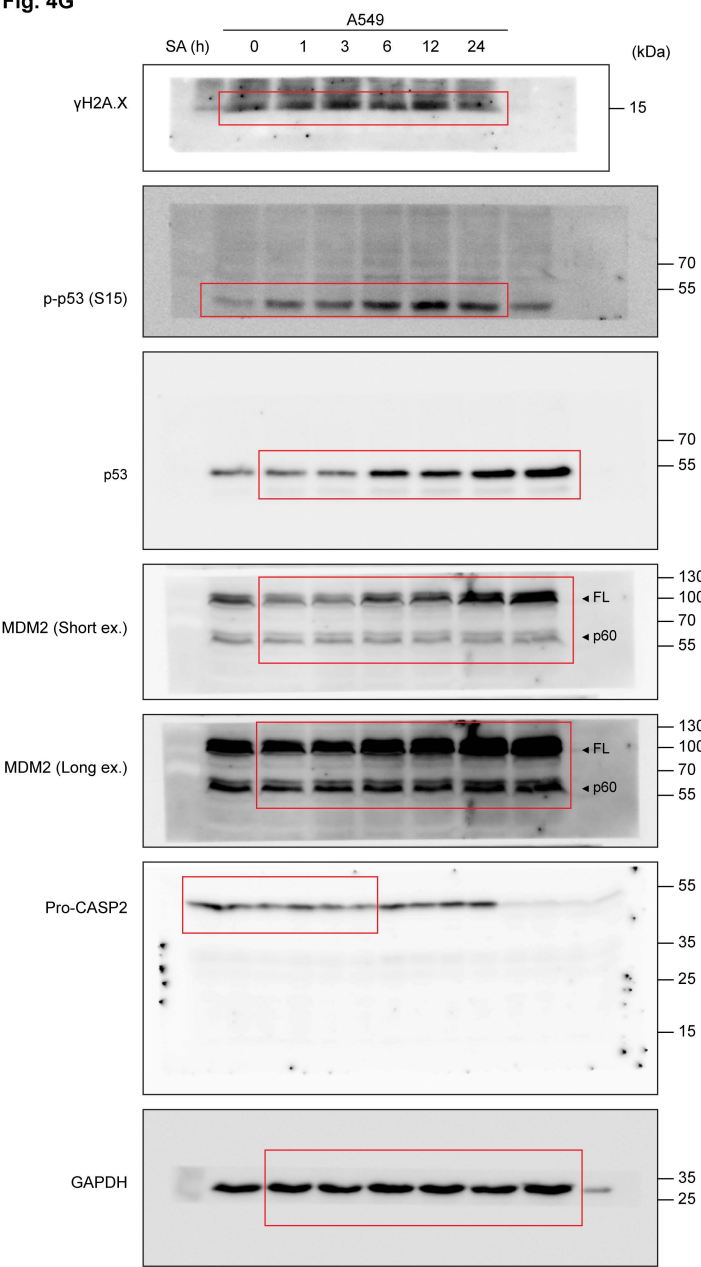

**Fig. 4D**

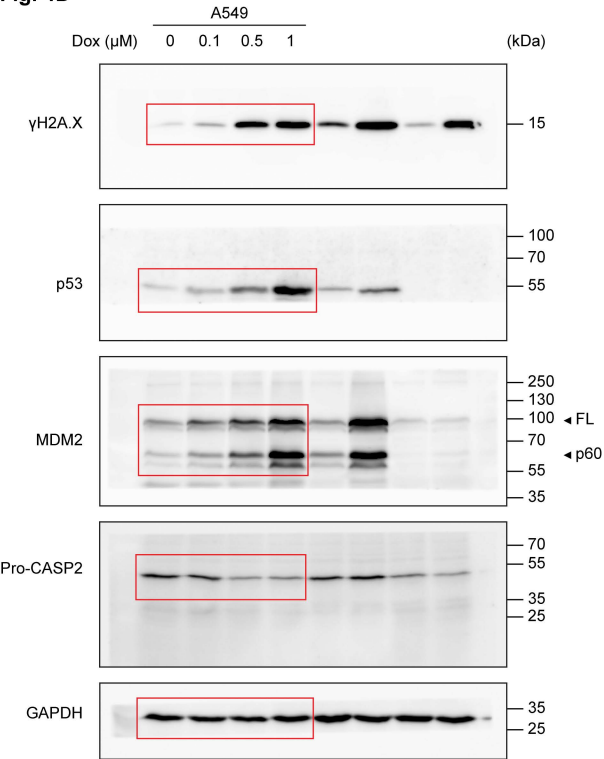

**Fig. 4H**

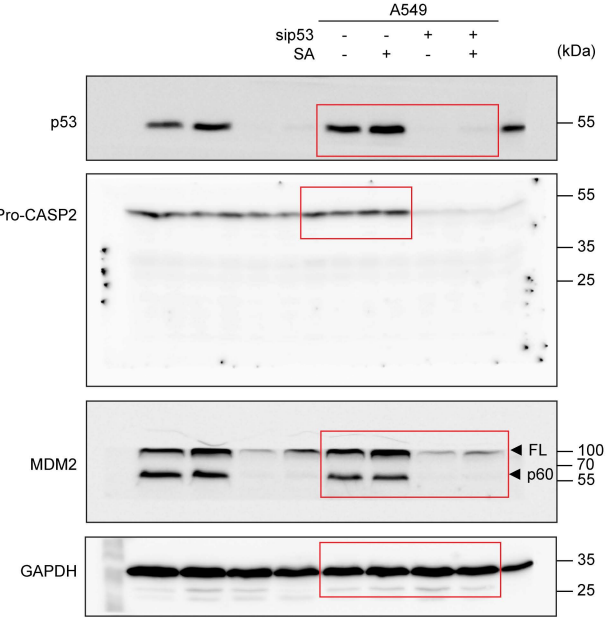

**Fig. 4I**

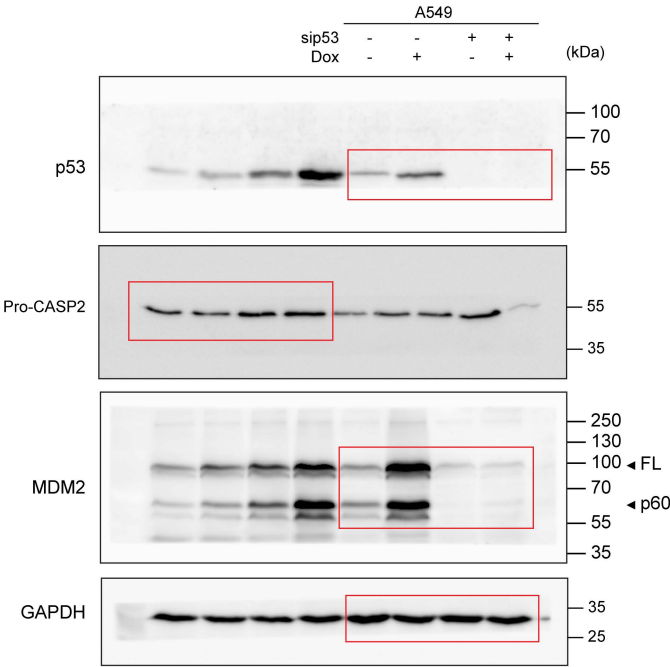

**Fig. 4K**

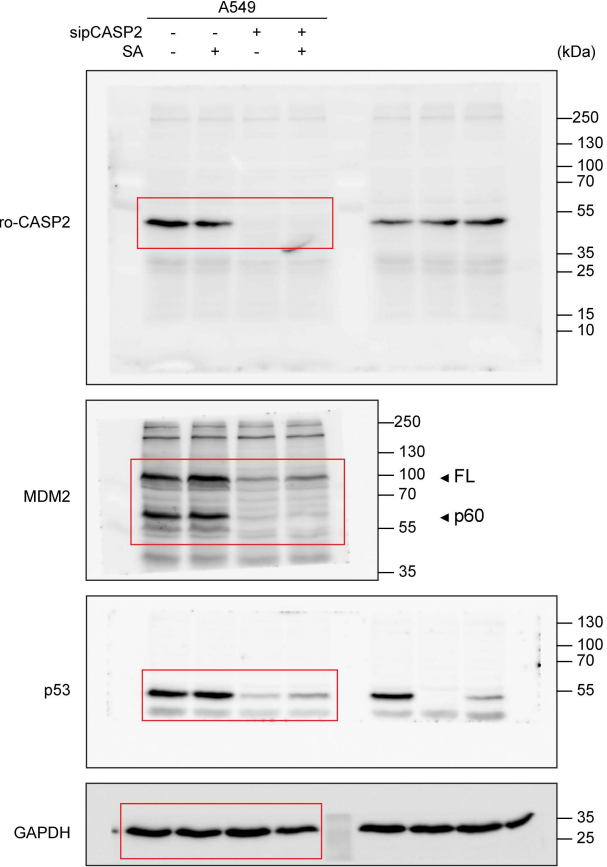

**Fig. 4L**

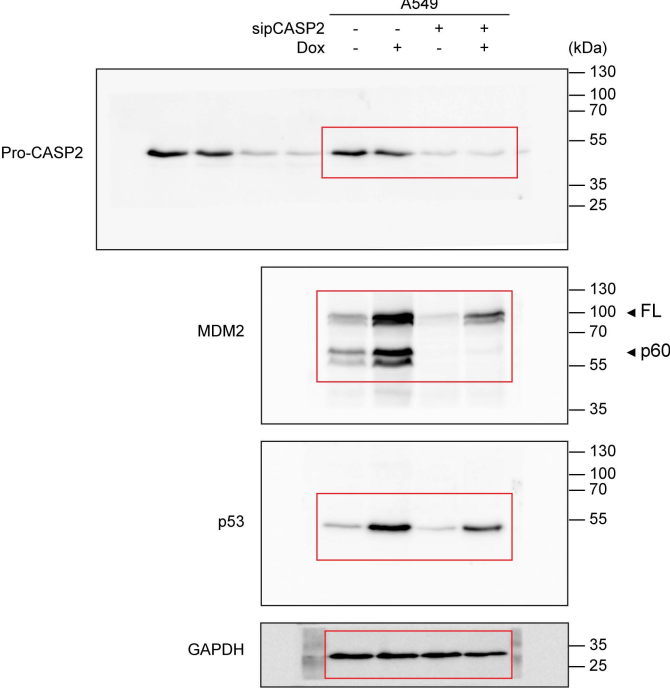

**Fig. 5D**

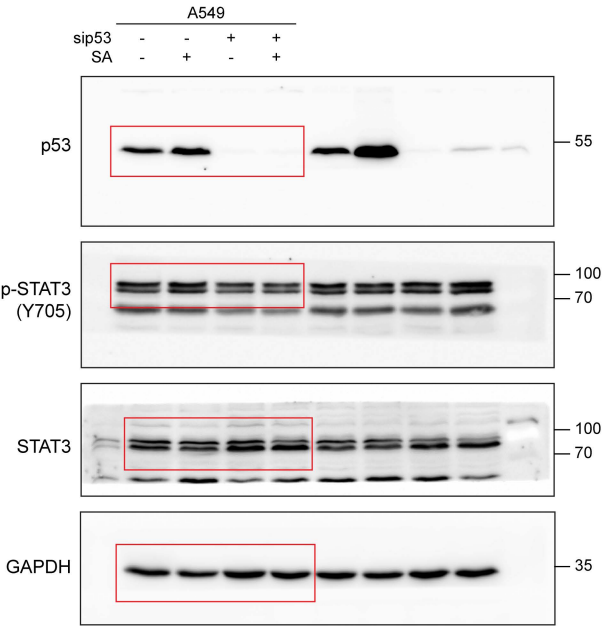

**Fig. 5G**

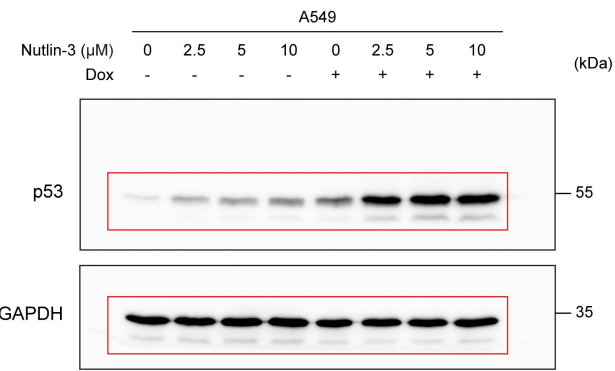

**Fig. 5L**

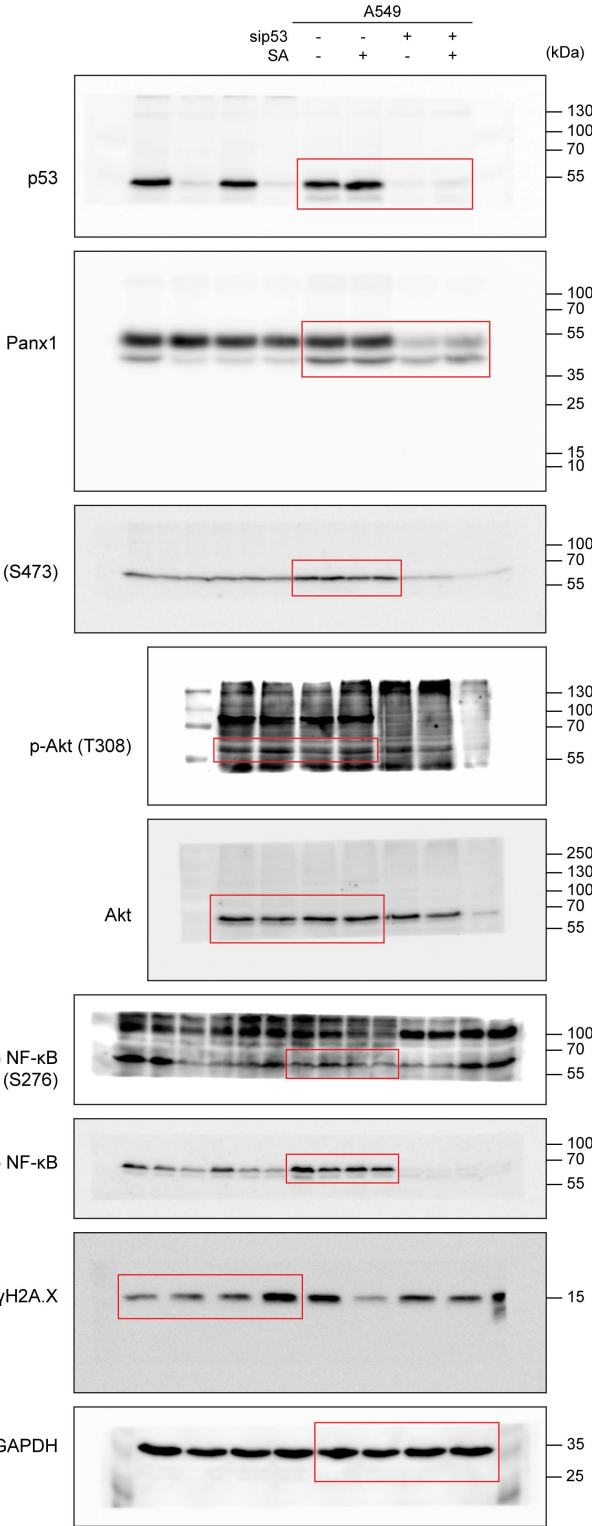

Supplement: Supplementary file 8 — Full and Uncropped Western Blots [file 41420_2023_1638_MOESM8_ESM.pdf]
